# Protein lifetimes in aged brains reveal a proteostatic adaptation linking physiological aging to neurodegeneration

**DOI:** 10.1126/sciadv.abn4437

**Published:** 2022-05-20

**Authors:** Verena Kluever, Belisa Russo, Sunit Mandad, Nisha Hemandhar Kumar, Mihai Alevra, Alessandro Ori, Silvio O. Rizzoli, Henning Urlaub, Anja Schneider, Eugenio F. Fornasiero

**Affiliations:** 1 Department of Neuro- and Sensory Physiology, University Medical Center Göttingen, 37073 Göttingen, Germany.; 2 German Center for Neurodegenerative Diseases, DZNE Bonn, Venusberg Campus 1, 53127 Bonn, Germany.; 3 Department of Clinical Chemistry, University Medical Center Göttingen, 37077 Göttingen, Germany.; 4 Bioanalytical Mass Spectrometry Group, Max Planck Institute for Multidisciplinary Sciences, 37077 Göttingen, Germany.; 5 Leibniz Institute on Aging—Fritz Lipmann Institute (FLI), 07745 Jena, Germany.; 6 Department of Neurodegenerative Diseases and Geriatric Psychiatry, University Hospital Bonn, 53127 Bonn, Germany.

## Abstract

Aging is a prominent risk factor for neurodegenerative disorders (NDDs); however, the molecular mechanisms rendering the aged brain particularly susceptible to neurodegeneration remain unclear. Here, we aim to determine the link between physiological aging and NDDs by exploring protein turnover using metabolic labeling and quantitative pulse-SILAC proteomics. By comparing protein lifetimes between physiologically aged and young adult mice, we found that in aged brains protein lifetimes are increased by ~20% and that aging affects distinct pathways linked to NDDs. Specifically, a set of neuroprotective proteins are longer-lived in aged brains, while some mitochondrial proteins linked to neurodegeneration are shorter-lived. Strikingly, we observed a previously unknown alteration in proteostasis that correlates to parsimonious turnover of proteins with high biosynthetic costs, revealing an overall metabolic adaptation that preludes neurodegeneration. Our findings suggest that future therapeutic paradigms, aimed at addressing these metabolic adaptations, might be able to delay NDD onset.

## Teaser

Physiological brain aging affects protein turnover, altering the stability of proteins linked to neurodegenerative diseases.

## INTRODUCTION

Aging is a prominent risk factor for neurodegenerative disorders (NDDs), including Alzheimer’s disease (AD), Parkinson’s disease (PD), amyotrophic lateral sclerosis (ALS), and Huntington disease (HD) (*1*). Analysis of brain protein levels in physiologically aged brain has revealed only minor alterations in protein abundances in the aged adult versus the young adult brain (*2*), reflecting differences in inflammation-related proteins or changes in proteasome and ribosome stoichiometry (*3*, *4*). This indicates that protein turnover, which regulates the equilibrium between protein synthesis and degradation, might be especially affected in aging and could lead to changes preluding neuropathology.

Findings concerning protein turnover changes in the aged proteome remain puzzling. Protein synthesis has been historically described as declining with age, although not all studies agree and often point to high organ and tissue variability [see (*5*, *6*)]. Protein degradation is also commonly described as compromised in aging (*7*, *8*). If both synthesis and degradation decline, lifetimes should increase and general turnover of proteins should be slower, possibly favoring the collapse of proteostasis networks and initiating the accumulation of potentially toxic proteins (*9*). While this general trend would explain the malfunctioning of macromolecules, protein turnover in different tissues has shown little or no overall changes in aged animals versus younger controls (*10*–*13*).

While results in invertebrate models suggest that proteostasis is essential for the survival of aging neurons (*14*, *15*), and that there is an age-related decline in protein turnover rates (*16*), in the aged mammalian brain an extensive quantitative analysis of protein turnover is currently lacking (*17*). Quantitative analysis would allow researchers to define the temporal coordination of proteins involved in aging and possibly in NDDs.

Our group has introduced an experimental workflow for the global quantification of protein lifetimes (*18*, *19*), which builds upon previous research on rodent turnover (*13*, *20*). Here, using this workflow, we obtained protein lifetimes in the aged brain cortex, in cerebellum, and in their synaptic fractions, aiming to provide cellular and subcellular information about changes in brain protein stability. We then compared protein lifetimes between young adult and aged mice addressing the changes observed during aging. We analyzed our results extensively with bioinformatics and revealed that the proteome in the aged brain is turned over at a slower rate (~20%). In addition, aging establishes an intrinsic alteration of the proteostasis network that specifically preserves proteins with high biosynthetic cost.

## RESULTS

**Fig. 1.**
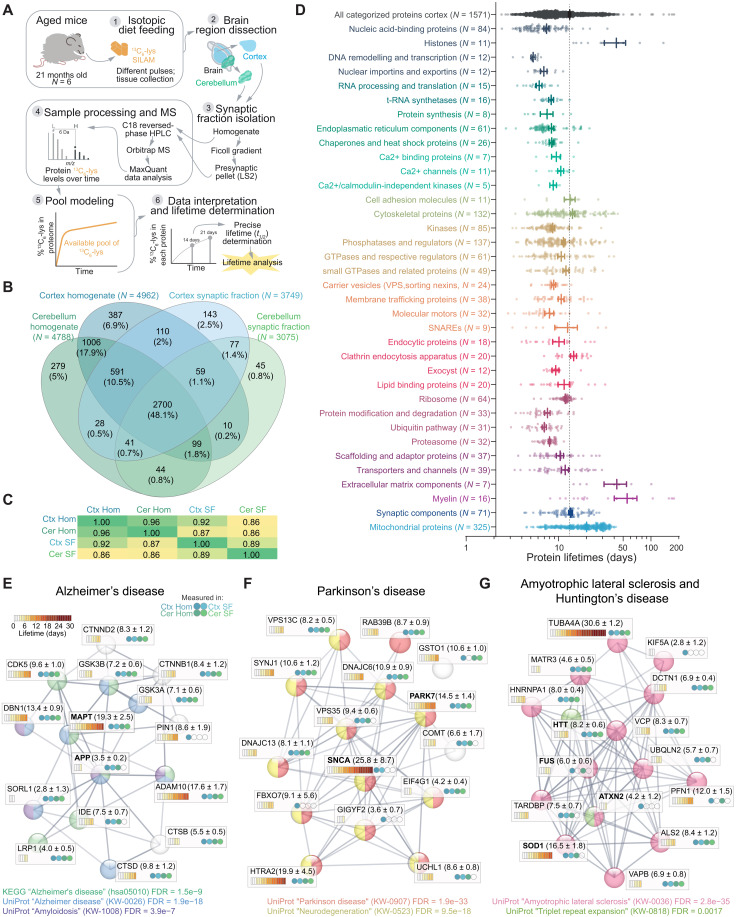
Precisely measured protein lifetimes in the cortex and cerebellum of aged mice. (**A**) Experimental workflow. Mice aged 21 months were metabolically labeled for 14 or 21 days, as previously described (*19*), and protein lifetimes were calculated for the brain cortex, cerebellum, and the respective synaptic fractions (*18*). (**B**) Venn diagram showing the LC-MS/MS protein identifications and their overlap in the four fractions. A total of 90,305 peptides (5354 protein groups) were identified in the homogenates, and 63,619 peptides (3947 protein groups) were identified in the synaptic fractions (see also fig. S1 and table S1). Numbers in parentheses show the number of proteins in each fraction. (**C**) Correlation matrix (Pearson’s *r*^2^) of calculated protein lifetimes from cortex and cerebellum, as well as respective synaptic fractions. (**D**) Lifetimes of 1571 proteins measured in cortex homogenate, subdivided in 36 categories, according to their organelle and/or functional affiliation [see (*19*) and table S1]. Each point corresponds to a single protein lifetime. Thicker lines indicate mean ± SEM for each category. The segmented line represents the average of all categorized proteins. (**E** to **G**) STRING networks (*77*) and graphical representation of protein lifetimes (expressed in days) in the brain of aged mice for a selection of proteins implicated in AD (E), PD (F), ALS, and HD (G). The four circles in each box represent in which sample type each lifetime was measured. The legends in the lower part of each panel formally clarify their association with the respective pathways. Significances are indicated as false discovery rates (FDRs) calculated for the specified pathways and reflect the relevance of the represented proteins for each neurodegenerative disease. Note a wide distribution of lifetimes within each pathology. NDDs are not generally coordinated by lifetimes.

To obtain protein lifetimes in the brain, we pulsed in vivo 21-month-old aged mice with a balanced and complete mouse diet where only the essential amino acid lysine (^12^C_6_-Lys) was substituted with the safe, nontoxic, and stable isotope of the same amino acid (^13^C_6_-Lys), enabling metabolic labeling with no impact on the animals’ health or metabolism (Fig. 1A) (*18*, *19*, *21*). Following a labeling period of 14 or 21 days, brains were dissected, synaptic fractions were enriched, 5619 protein groups were identified with liquid chromatography–mass spectrometry (LC-MS) (Fig. 1B), and ^13^C_6_-lys/^12^C_6_-lys ratios [i.e., heavy versus light ratios (H/L)] were determined for 4807 proteins across four sample groups (cortex, cerebellum, and their respective synaptic fractions) containing three biological replicates per labeling period and tissue (fig. S1A).

To obtain the precise protein half-life (*t*_1/2_; referred to in this work as lifetime) from the labeling results, we accounted for the reuse of lysines from the degradation of proteins (*18*). Acquiring lifetimes with this workflow is time consuming but very robust and reproducible (*18*). It relies on the ratios independently obtained from heavy- and light-labeled peptides, and no channel boosting or extensive data processing steps are necessary, in contrast with other more recent workflows (*22*). Following the fitting of the H/L ratios across six biological replicates (three per labeling period, 14 and 21 days) and the unlabeled “0-day” values, we obtained reliable measures and their confidence intervals (referred to as c1 and c2 for lower and upper bound, respectively, as detailed in table S1) of 3769 protein lifetimes, in the aged brain cortex, in the cerebellum, and in their respective enriched synaptic fractions (fig. S1, B to E, and table S1).

The average lifetime of the aged cortex proteome calculated across 3252 proteins is 11.41 ± 0.16 (SEM) days, and the lifetime of the synaptic proteome enriched in the synaptic fraction is significantly increased by ~20% (unpaired *t* test, *P* < 0.0001; fig. S1, D and E) similar to the young adult brain (*19*, *23*). The same ~20% increase in lifetime for the synaptic fraction is observed in the cerebellar extracts (unpaired *t* test, *P* < 0.0001; fig. S1, D and E) and likely reflects a subcellular difference of the proteostatic balance. We considered specifically the direction of the changes in proteins whose lifetimes differ in the aged synaptic fractions compared to the respective aged homogenates in both the cortex and cerebellum (1143 proteins; fig. S2). This analysis revealed that, as in the case of the young adult brain (*19*), on average, most proteins have longer lifetimes in synaptic fractions (751 longer-lived proteins in the synaptic fraction versus 392 relatively shorter-lived in these fractions, corresponding to ~66% stabilized proteins among those considered here). Using synaptic gene ontologies (SynGOs) (*24*), we found an enrichment of the proteins that are preferentially stabilized in presynaptic structures, such as active zone and synaptic vesicle components (fig. S2C and table S1). By contrast, among the shorter-lived proteins in synaptic fractions, there are relatively more postsynaptic density proteins (fig. S2D and table S1), including the PSD-95 binding proteins Dlg-associated protein 1 and 4 (Dlgap1 and Dlgap4) or Shank2.

Generally, as also observed in the young adult rodent cortex (*20*, *25*) and in other tissues (*26*), protein lifetimes have a large variation and are log-normal distributed, as confirmed by the fact that all datasets pass an Anderson-Darling test, indicating that they can be approximated with a normal distribution after calculation of log_10_. These “log-normal” distributions arise from similar events that give rise to a “normal” distribution, with the caveat that the process at their bases might be influenced by the addition of small percentage changes that become additive on a logarithmic scale and/or by multiplications and divisions of positive variables. Several biological response events in time are well approximated by a log-normal distribution, as in the case of pharmacokinetic variables (*27*).

Moreover, as also observed before, histones, extracellular matrix proteins, and myelin components are among the longest-lived proteins in the aged mice cortex, while transcription factors and proteins involved in mRNA processing and translation are short-lived (Fig. 1D and table S1; see fig. S3 and table S2 for a comparison of the here acquired lifetimes to previous works). Our lifetimes are perfectly in line with a previous work on aged mouse tissues, including brain, which was only focusing on proteins for the respiratory chain (*6*). This is quite remarkable, especially since two different labeling technologies and analysis workflows were used, confirming the robustness of these methods and the reproducibility of lifetime measures across studies (fig. S3 and table S2).

Next, we focused on the lifetimes of proteins linked to NDDs (Fig. 1, E to G; for selection details, please see Materials and Methods). We observed no statistically significant differences between average lifetimes of proteins involved in specific NDDs (averages: AD = 8.8 ± 1.2 days, PD = 10.5 ± 1.5 days, and ALS = 9.1 ± 1.5 days; not significantly different from each other). The lifetimes measured for these proteins mostly correspond to the lifetimes of proteins that belong to similar functional classes.

For example, the microtubule-associated protein tau (MAPT) has a lifetime of 19.3 ± 2.5 days, which is similar to other proteins associated with the microtubule cytoskeleton and coincides with what has been measured in the human brain by a targeted approach (23 ± 6.4 days) (*28*). Other proteins associated with AD, such as the amyloid precursor protein (APP) and the sortilin-related receptor (SORL1), have short lifetimes (3.5 ± 0.2 and 2.8 ± 1.3, respectively; Fig. 1E). These results reflect similar observations for mRNA and protein abundances since they span different orders of magnitude (*29*, *30*). These absolute lifetimes can be useful for planning experiments aimed at observing effects on the modulation of gene expression in animal studies of NDDs and define the appropriate time window for experiments in aged mice (see table S1 for detailed lifetimes). As an example, following inactivation of the superoxide dismutase gene (*SOD1*), it would be necessary to wait 16.5 ± 1.8 days to observe a decrease of 50% of the protein in the aged mouse brain.

**Fig. 2.**
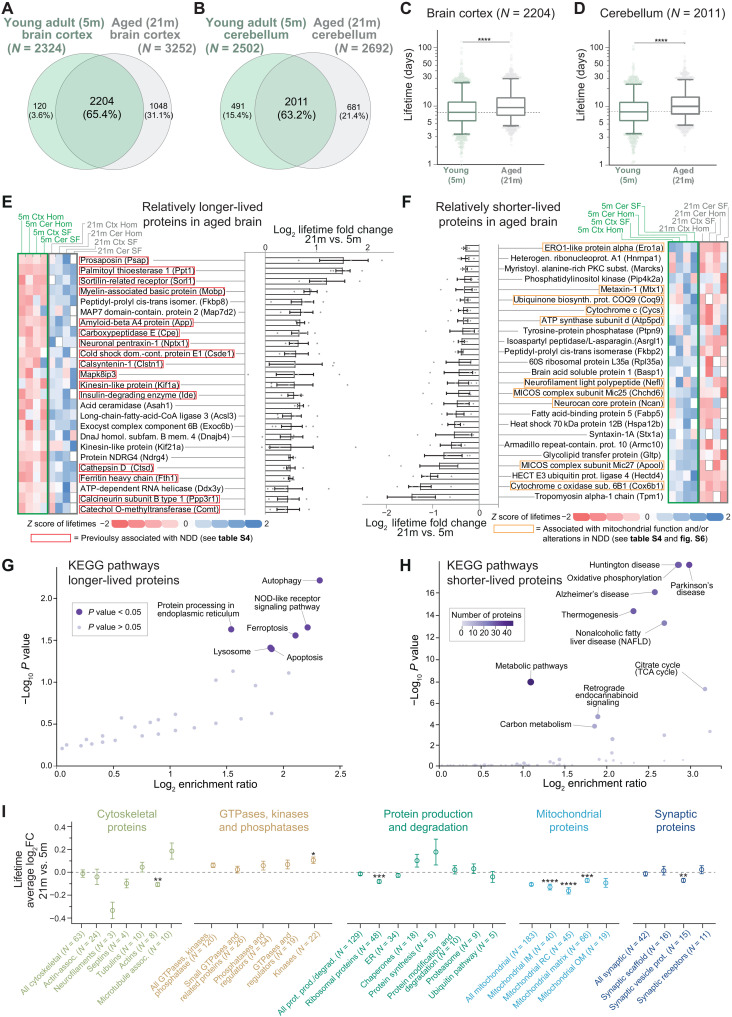
Specific lifetime changes in the aging brain. (**A** and **B**) Venn diagrams of lifetimes measured here [“aged mice”; 21 months (21m)] or previously published [“young adult mice”; 5 months (5m); (*19*); see also table S3]. (**C** and **D**) Comparison of lifetimes in cortex (C) or cerebellum (D) of 5- and 21-month-old mice (nonparametric Wilcoxon matched-pairs signed-rank test, ****P ≤ 0.0001; boxplots represent median, 25th to 75th percentile, with whiskers showing 5th to 95th percentile). Turnover is significantly lower in both cases. (**E** and **F**) Summary of 50 proteins whose lifetime after rescaling is either relatively longer-lived (rLL) (E) or relatively shorter-lived (rSL) (F) in at least three of four turnover datasets analyzed here (brain cortex, cerebellum, synaptic cortical, and synaptic cerebellar fraction; see also table S3). Heatmaps show lifetimes color-coded as *z* scores. Gray boxes, not measured. Log_2_ fold change (log_2_FC) summarizes ratios of protein lifetimes of 21- versus 5-month-old mice (±SEM). Red boxes in (E) indicate proteins implicated in NDDs (see table S4). (**G** and **H**) Kyoto Encyclopedia of Genes and Genomes (KEGG) pathway analysis of rLL in aged mice in all four datasets (G) (*N* = 72) or always rSL in aged mice (H) (*N* = 128), showing lifetime changes of at least >10% (for details, see table S3 and fig. S6). Several mitochondrial proteins linked to NDDs appear relatively shorter-lived in aged mice as summarized in (H) and in fig. S6B. (**I**) Proteins either rLL or rSL in aged mice according to functional affiliation [see (*19*) and table S5]. Mean ± SEM of the log_2_FC (21 months versus 5 months). Proteins per category are indicated in parentheses. Significance against the consistently changed lifetimes was calculated using Brown-Forsythe and Welch ANOVA followed by Dunnett multiple comparison correction (*P ≤ 0.05, **P ≤ 0.01, ***P ≤ 0.001, and ****P ≤ 0.0001). Only significant categories are reported (for the remaining list, see fig. S7).

We then compared the lifetimes of the 21-month-old aged brain determined in this study with those that we previously obtained from young adult brains (5-month-old) (*19*), where the workflow, the instrumental setup, and the analysis were identical, facilitating a robust and meaningful comparison of ~2000 proteins both in the brain cortex and cerebellum (Fig. 2, A and B) and in their respective synaptic fractions (fig. S4, A and B, and table S3). In general, all proteins in the aged brain have longer lifetimes compared to the young adult brain (Fig. 2, C and D, and fig. S4, E and F; Wilcoxon test, *P* < 0.0001). In detail, proteins live ~20% longer in the aged adult compared to the young adult brain (with medians measured across the whole proteome of 21.7% in the cortex and 24.3% in the cerebellum at 21 months versus 5 months). Reduced lifetimes might be influenced by food consumption, but we excluded this possibility, as food intake was not significantly different between aged and young adult mice (fig. S5). This is also in line with the observations showing that in other tissues, such as liver and muscle, protein turnover is mostly unchanged or it is changed in an opposite direction in aged mice versus younger mice (*10*–*12*).

After observing this general difference, to be able to further analyze differences in the relative change of protein lifetimes, we proceeded to account for the systematic decrease of proteome turnover and rescaled the turnover measurements accordingly. Following linear median rescaling, lifetimes were not significantly different (fig. S4, A, B, G, and H), indicating that the overall proteome is affected by this change and that the process can be summarized with a simple linear relationship.

Thus, to reveal protein-wise changes in the biosynthetic priorities of the aged proteome, we next sought to examine which proteins, besides the general lengthening of protein lifetimes in the aged brain, are either relatively shorter-lived (rSL) or relatively longer-lived (rLL) compared to the young adult brain. For this, we calculated the ratios between the rescaled protein lifetimes in the aged brain versus the protein lifetimes observed in the young adult brain in each of the four sets of samples, expressed as logarithm of the fold change (log_2_FC; table S3). These values represent the extent of lifetime change between aged and young brain in the cortex, in the cerebellum, and in their respective synaptic fractions. We also reasoned that, besides tissue- and cellular compartment–specific differences, if there are systematic changes in the aged proteome, these should be conserved across these four datasets. We then assessed the averaged changes, considering only proteins for which lifetimes were changed in the same direction in at least three of the four turnover datasets (including brain cortex, brain cerebellum, synaptic cortical fraction, and synaptic cerebellar fraction), corresponding to 991 proteins (table S3). We concentrated on the two extremes of these changes, since these would reflect the most pronounced changes in relative lifetimes. The 25 proteins that showed either the most pronounced increase or decrease in lifetimes from this analysis are summarized in Fig. 2 (E and F) (rLL and rSL, respectively; for the entire list, see table S3).

Unexpectedly, among the top 25 rLL proteins, several have been previously associated with NDDs (Fig. 2E and table S4), including the amyloid precursor protein (APP), sortilin-related receptor (SORL1), the lysosomal proteolytic enzyme cathepsin D (CTSD), ferritin heavy chain 1 (FTH1), the neuroprotective carboxypeptidase E (CPE), prosaposin (PSAP), and calsyntenin 1 (CLSTN1; see table S4 for a complete list with additional references). By studying rSL proteins, we noticed that several of these were specifically implicated in mitochondrial function and metabolism (Fig. 2F), which are known processes disrupted in NDDs (*31*).

Besides our initial observations, which were guided by the literature, formal pathway analysis confirmed that, on one side, rLL proteins in the aged brain are associated with pathways underlying aging and neurodegeneration such as oxidative stress, autophagy, ferroptosis, lysosome function, and translation efficiency (Fig. 2G, fig. S6A, and table S3) (*4*, *8*, *32*–*34*). On the other side, Kyoto Encyclopedia of Genes and Genomes (KEGG) pathway analysis of the rSL proteins also revealed a strong direct association to NDDs such as HD, AD, and PD with high significance level (Fig. 2H, fig. S6B, and table S4). This is due to a high proportion of components of mitochondrial complexes I and V that are altered in neurodegeneration, which show a decreased function in the aged mouse brain (*35*).

An additional analysis on manually curated protein categories (*19*) mirrored these changes, with mitochondrial proteins having consistently reduced lifetimes in several subclasses (Fig. 2I and table S5). Notably, although we observed a positive correlation between changes in protein levels and changes in protein turnover, analyses of protein abundance changes in aged versus young adult mice performed in parallel with our lifetime measurements did not show significant changes of these mitochondrial subclasses (fig. S8 and tables S1 and S5). This is not surprising since level changes are small in the aged brain (*3*), and specifically, mitochondrial protein levels have complex trajectories during the lifespan of mice (*36*). Instead, turnover measures provide a direct snapshot of proteome dynamics at a specific age (*18*). To understand in a more general perspective the relationship between changes in protein lifetimes and protein abundances during aging, we took into consideration (i) proteins with increased lifetime and increased abundance, (ii) proteins with increased lifetimes but decreased abundance, (iii) proteins with decreased lifetimes and decreased abundance, and (iv) proteins with decreased lifetimes but increased abundance (fig. S8B). This analysis revealed, for example, that proteins with decreased lifetimes and decreased abundance are more numerous in the aging datasets, although the interpretation of these results requires a careful evaluation (for a detailed discussion of these results, refer to text S1 and see fig. S8B).

We also sought to understand whether protein lifetimes were differentially changed in different cellular subpopulations. However, while protein abundances measured in bulk might depend on the composition of neurons and glia and their age-related decrease or increase (*37*, *38*), the lifetime of proteins within these cell populations is likely not affected, unless the turnover of the entire cell population is strongly changing at the time of labeling. This analysis revealed no gross changes in protein lifetimes between aged and young adult brains for proteins previously reported to be specific for microglia, astrocytes, neurons, or oligodendrocytes [following a previous cell type–specific protein classification (*39*); fig. S7E and table S6] and indicates that at the time of labeling there are no differences in the proliferative rates of these cells. This is in agreement with previous observations indicating astrogliosis in the aged brain, also mirrored by the level increase of structural proteins such as GFAP (*40*), and suggests that at 21 months of age glial cells already peaked in these mice.

**Fig. 3.**
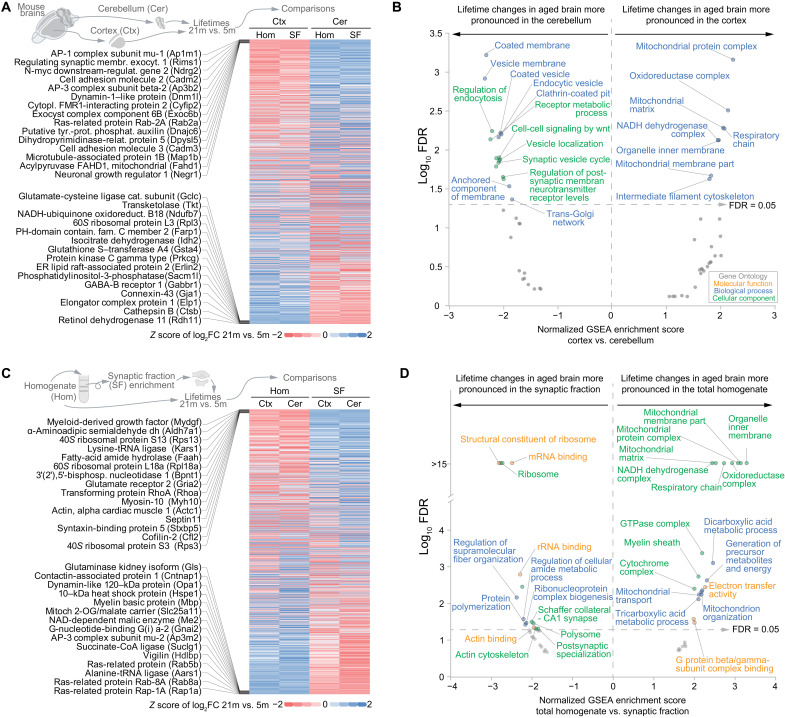
Cell type, regional, and synaptic specificities of protein lifetime changes in the aged versus the young adult brain. (**A**) Heatmap summarizing lifetime changes for 1143 proteins in the aged brain with respect to the young adult brain [representing the fold change expressed as log_2_ (log_2_FC) between 21- and 5-month-old mice as a *z* score], showing modifications in lifetimes that in aging affect more specifically either brain cortex or cerebellum. Because of space limitations, only the 15 rSL proteins (top) or the 15 rLL proteins in the cortex versus the cerebellum (bottom) are shown (for a detailed list, see table S6). (**B**) Gene set enrichment analysis of (A), summarizing the molecular function, biological process, and cellular component of the gene ontologies (GOs) significantly enriched with an FDR of <0.05 (for the whole results, see table S6). (**C** and **D**) Comparison [represented as in (A) and (B)] between lifetime changes in the total homogenate and the respective enriched synaptic fractions that were obtained as previously described (*19*).

**Fig. 4.**
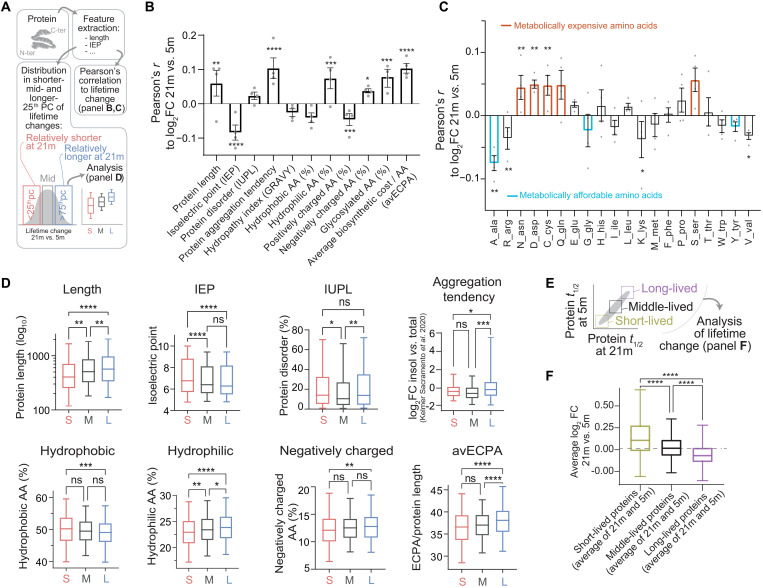
Correlations between protein features and protein lifetime changes reveal a generalized shift of metabolic resources in the aged brain. (**A** to **D**) Analysis of biochemical properties linked to lifetime changes in aged versus young brain. We obtained biochemical properties of proteins (see Materials and Methods and table S7), and we then measured (i) their correlations to change of lifetime in aged mice versus young mice (B) and (ii) difference for each parameter in three protein subgroups defined as the rSL quarter of the aged proteome (<25th percentile), the middle quarter (37.5th to 67.5th percentile), and the rLL quarter (>75th percentile) (D). Pearson’s coefficients (B) show correlations, where bars represent mean ± SEM and points are changes in the individual datasets (Pearson’s *P* values: *P ≤ 0.05, **P ≤ 0.01, ***P ≤ 0.001, and ****P ≤ 0.0001). (C) Pearson coefficients summarizing the correlations of lifetime log_2_FCs to amino acid composition of proteins. Bars represent mean ± SEM, and points are changes in the individual datasets (Pearson’s *P* values). Amino acids in orange (expensive) have a positive correlation with lifetime change, while cyan ones (affordable) show an opposite trend. The percentile analysis (D) reinforces these findings (significance was calculated with either one-way ANOVA and Tukey multiple comparison correction or Brown-Forsythe and Welch ANOVA test with Games-Howell multiple comparison correction if SDs were significantly different between groups). (**E**) We considered proteins, on average, either short-lived, middle-lived, or long-lived. (**F**) Within these groups, we calculated average lifetime change in aged brain versus young adult brain (log_2_FC, 21 months versus 5 months). Short-lived proteins tend to live relatively longer in the aged brain than in the young adult brain, while longer-lived proteins live shorter in the aged brain, pointing to a compression of proteome lifetimes with age (see also fig. S7). Boxplots: median, 25th to 75th percentile; whiskers, 5th to 95th percentile.

Having analyzed the overall changes across all datasets, we tested whether we could find regional or subcellular differences in the lifetime changes. For this purpose, we compared the log_2_FC of the lifetimes between aged and young adult mice and checked which proteins showed more prominent lifetime changes either in the cortex or in the cerebellum (see Fig. 3 and table S6). This analysis showed that in synaptic vesicle proteins, which are rSL in the aged brain (Fig. 2I), the lifetime change is more pronounced in the cerebellum, where the endocytic machinery also seems to be specifically affected (Fig. 3B, left-side part of the graph). In contrast, age-associated alterations in lifetimes of mitochondrial proteins were most prominent in the cortex (Fig. 3B, right-side part of the graph). These regional differences might reflect a higher relative number of neurons with elevated synaptic activity rates in the cerebellum, whereas the cortex, which is generally more affected by NDDs (*41*), shows more pronounced changes in mitochondrial pathways and respiration. We then performed an analysis of lifetime changes in the synaptic fractions versus the total cellular homogenate (Fig. 3, C and D, and table S6). This revealed a preferential change in the lifetime of ribosomal proteins localized at synapses in the aged brain compared to the young adult, possibly suggesting a specific altered local transcription at synapses in the aged brain (Fig. 3D, left-side part of the graph). On the contrary, the age-dependent lifetime changes in mitochondrial proteins are not specific for synapses but rather affect the mitochondria in the whole-brain homogenate (Fig. 3D, right-side part of the graph). α-Synuclein (SNCA), which was found in the cortex (see Fig. 1F), showed a lifetime extension only in the synaptic fraction, indicating a preferential control of SNCA turnover at the synapse in the aged brain. Last, we explored the correlations between biochemical protein parameters and the changes in protein stability in the aged brain (Fig. 4, A to F).

We thus proceeded by collecting biochemical parameters such as protein length, isoelectric point, intrinsic disorder, and other amino acid properties (Fig. 4A and table S7). We then analyzed the Pearson’s correlations and the significances of these parameters to the average relative fold change of the lifetimes between aged and young mice (Fig. 4B). We also considered the distribution for each parameter in three protein subgroups defined as rSL proteins in the aged brain versus the young adult (<25th percentile), middle-lived proteins (corresponding to the 37.5th to 67.5th percentile), and rLL proteins (>75th percentile; Fig. 4D). Even if correlations are modest, several of these were significant and lead to noteworthy observations.

First, we tested whether biochemical features correlate to changes of protein turnover during aging. Proteins with higher isoelectric point tend to live relatively shorter in the aged brain (Fig. 4, B and D). This result is reinforced by a significant negative correlation with positively charged amino acids (which also concur to define the isoelectric point). Proteins with a low isoelectric point are preferentially localized and degraded in lysosomes (*42*, *43*); hence, a possible explanation is that following the progressive loss of lysosomal proteolytic activity observed during aging (*34*), these proteins become rLL.

Second, we tested whether rLL proteins tend to be found more predominantly in the aggregate-enriched cellular fraction, which could imply that they are less efficiently degraded. For this, we compared our data with the recent dataset of insoluble-enriched proteins in the aged mouse brain (*4*) and found a positive correlation of lifetime change and aggregate-enriched fraction. We observed that rLL proteins also have a slight tendency to be more disordered [IUPL (intrinsically unstructured large proteins); Fig. 4, B and D]. We also noticed that the number of glycosylated residues is positively correlated with the shift in lifetimes, in line with previous reports observing alterations of glycosylated proteins in aging and providing a possible link to neurodegenerative disease (*44*–*47*).

Third, we tested whether metabolically “more expensive” proteins are preferentially stabilized in the aging brain, which would be in line with bioenergetic changes in the aging brain (*48*, *49*). As an example, longer proteins (with higher molecular weight) use more amino acids per chain and thus require more energetic resources. We observed a positive correlation for protein length (Fig. 4, B and D). We additionally considered the average energy cost per amino acid (avECPA) for protein biosynthesis (*50*); we also found in this case that turnover for proteins with especially expensive components (e.g., cysteine, aspartate, and asparagine; Fig. 4C) is decreased in the aged brain, thus reducing energy expense.

Fourth, it is tantalizing to speculate that aging leads to general unbalanced homeostatic regulation, thus preferentially affecting exceptionally short- or long-lived proteins, since these would be under greater proteostatic pressure, requiring specific mechanisms to be either degraded fast or preserved for longer times (*51*). In simple terms, this would lead to a compressed range of the lifetimes observed.

To test this hypothesis, we first measured the correlation between the lifetime change in the aged brain and the average protein lifetimes (measured as an average of lifetimes in the aged and young adult brain). We observed a negative correlation (Pearson’s *r* = − 0.1583; *P* < 0.0001), indicating that rSL proteins are longer-lived while rLL proteins are shorter-lived and supporting a compression of lifetimes in the aged brain. Next, we formally proved that, when comparing the protein lifetimes between the aged and the young adult brain (Fig. 4E), proteins that are short-lived in both datasets are, on average, rLL in the aged brain, while vice versa proteins that are long-lived in both datasets are, on average, rSL in the aged brain (Fig. 4F).

To exclude the possibility that these observations are due to a bias originating from our analysis workflow, we also checked the original H/L (^13^C_6_-lys/^12^C_6_-lys) ratios and found that there is an overall bidirectional change of protein labeling efficiency, so that even in absolute terms short-lived proteins are less labeled in the aged brain than in the young adult (and thus longer-lived), while the long-lived ones are more labeled (and thus shorter-lived; fig. S9, B and C). This opposite bidirectional change confirms that aging favors general deregulation of protein homeostasis that affects the balance of the entire proteome. Last, we also considered whether regional and synaptic changes in protein lifetimes could be explained in the light of biochemical properties, and we found that there are some significant correlations that are preferential for synaptic fractions or for the brain regions analyzed (cortex and cerebellum; for details, see fig. S10 and text S2).

## DISCUSSION

Here, using stable isotope metabolic labeling and advanced data analysis (*18*), we provide the first quantitative extensive examination of protein lifetimes in the aged mammalian brain, complementing previous findings on proteins of the respiratory pathway (*6*). Our work represents a large resource for the community and expands the constellation of proteomic technologies available for the study of brain aging (*52*–*54*). Our observations indicate that protein turnover in the aged brain is overall ~20% slower than in the young adult, settling an open question in the field and stimulating the development of future research that will address how this is achieved at the molecular level. Strikingly, differential lifetime analysis between aged and young brain showed that aging alters a subset of proteins related to NDDs. Such an alteration of NDD protein lifetimes was previously unknown and occurs in different classes of proteins that are either rLL or rSL in the aged brain versus the young adult brain.

In detail, across the rLL proteins in the aged brain, there are known players of NDDs such as APP and SORL1. Other rLL proteins are linked to oxidative stress, autophagy, ferroptosis, and lysosome function. We observed that most neurodegeneration-related rLL proteins exert neuroprotective functions, most prominently in AD. AD is characterized by the pathological aggregation of amyloid-β and tau. Amyloid-β peptide is generated from the APP by the concerted action of two proteases on the expense of the production of neuroprotective and neurotrophic soluble APPα fragment (*55*). The endocytic receptor sortilin-related receptor SORL1 binds to APP and regulates its intracellular trafficking and amyloidogenic processing (*56*). SORL1 loss-of-function mutations have been linked to familial AD and increased production of amyloid-β (*57*). Calsyntenin 1, which is also among the rLL proteins, is required for APP transport through the axon, and its loss results in altered APP processing and increased amyloid-β production (*58*). The insulin-degrading enzyme (IDE), another rLL protein, cleaves amyloid-β peptide in the brain and has a neuroprotective function (*59*), similar to the lysosomal protease cathepsin D (also an rLL protein), which degrades intracellular amyloid-β and tau (*60*). Apart from its role in AD, cathepsin D also cleaves prosaposin to generate saposins A to D. Loss of prosaposin in neurons was recently shown to result in accumulation of lipofuscin, oxidative stress, and cell death due to ferroptosis (*61*). The rLL protein FTH1, which is highly expressed in oligodendroglia and transferred to neurons, prevents ferroptosis (*62*). Last, loss-of-function mutations in the rLL protein palmitoyl thioesterase-1 (PPT-1) result in neuronal ceroid lipofuscinosis, a neurodegenerative lysosomal storage disorder (*63*). Together, a notably high number of rLL proteins fulfill important neuroprotective functions, and their loss or altered functionality is implicated in NDD. Although it would be interesting to speculate that these proteins become rLL because they are less efficiently exchanged and thus lose some of their protective activity, we do not yet know whether this is true and future studies will be required to explore this avenue.

Among the rSL proteins in the aged brain, we find several components of mitochondrial complexes. These include proteins upholding mitochondrial structure organization, like metaxin 1 (MTX1) and components of the MICOS complex in the mitochondrial inner membrane (such as APOOL and CHCHD6). Further, we find several proteins involved in cellular respiration, like ubiquinone biosynthesis protein COQ9, mitochondrial adenosine triphosphate synthase subunit delta (ATP5F1D), cytochrome c (CYCS), and cytochrome c oxidase subunit 6b1 (COX6B1). In general, many of these mitochondrial proteins are known to be long-lived (*19*, *64*), and the fact that they tend to be shorter-lived in the aged brain compared to the young adult brain is in agreement with the overall compression of lifetimes that we have observed here (see discussion below). Recently, dopaminergic neurons with disrupted function of mitochondrial complex I have been described to be remarkably plastic in terms of energy production and physiology, strengthening neuronal survival, but to the detriment of dopamine release, ultimately inducing parkinsonism (*65*). As highlighted by this complex paradigm, the link between mitochondrial homeostasis and neurodegeneration is multifaceted. While failing mitochondrial function and energy production are well-known phenomena in aging and NDDs (*35*, *66*), we can only speculate on the actual functional outcome of relative shorter lifetimes in mitochondrial proteins found here. Faster turnover could represent another layer of plasticity, a response to physiological aging and changing energy demands of the brain; it could, however, also drive mitochondrial dysfunction. Future mechanistic studies will be required to elucidate the details of protein turnover and its link to mitochondrial and cellular function.

To reveal brain- and subcellular-specific changes in protein turnover, which might reflect molecular changes (*67*), we analyzed cortex, cerebellum, and their respective synaptic fractions. In our analysis, it appeared that mitochondrial changes were prominent in the whole lysate and more pronounced in the brain cortex than in the cerebellum, suggesting a specific alteration of mitochondrial proteostasis in the aged cortex, in line with previous findings (*35*). At variance, a closer look at the changes in the turnover of synaptic datasets revealed that ribosomal proteins are especially different in the synaptic fraction of aged mice. Since protein turnover can be indicative of changes in the functional states of proteins (*67*), and synaptic ribosomal translation is an important determinant of brain plasticity (*68*, *69*), the differences that we observe might indicate functional changes in the local translation machinery at aged synapses. In contrast to what has been recently observed in knockin mouse models of AD (*70*), turnover of presynaptic proteins in physiologically aged mice does not show marked changes, suggesting that once neurodegeneration becomes prominent, additional pathological mechanisms are probably initiated.

Among the limitations of our work, one should mention the bias toward detecting and measuring proteins that are more abundant, which is common in the MS field (*71*). In the future, technologies that provide more comprehensive proteome coverage will reveal changes for proteins that might not have been detected in our study. One additional obvious limitation of our study is that we do not know to which extent the measurements of protein lifetimes in mice relate to humans. Nevertheless, with the appropriate corrections, global patterns of brain-specific mRNA and protein levels for orthologous genes are well conserved between human and mouse (*72*, *73*), and this is also likely the case for protein lifetimes (*74*). In any case, we have identified here a series of novel protein targets, which might be linked to NDDs and will need to be assessed in the human context.

With a database containing thousands of protein turnover measures during aging, we could explore the correlations between biochemical protein parameters and changes in protein stability. We reveal an overall proteostatic change reflecting a reprioritization of bioenergetic costs, which preserves the more expensive proteins in the aged brain while replacing more readily proteins that are metabolically less expensive, leading to an overall compression of protein lifetimes and an overall change in the balance of the entire proteome. This is in line with the recent theory that alterations observed in NDDs might be linked to proteomic cost minimization (*49*). Overall, our work constitutes the foundation for future studies on this subject that will address more specifically the role of prepathological alterations occurring during “healthy brain aging” and test metabolic approaches with therapeutic potential to hinder the initiation and the progression of NDDs.

## MATERIALS AND METHODS

### Mice and SILAC labeling

All mouse experiments were approved by the local authority, the Lower Saxony State Office for Consumer Protection and Food Safety (Niedersächsisches Landesamt für Verbraucherschutz und Lebensmittelsicherheit). Aged (20 months) wild-type male C57BL/6JRj mice were purchased from Janvier Labs. SILAC (stable isotope labelling by amino acids in cells) diets L-^12^C_6_-lysine (K0) and L-^13^C_6_-lysine (K6) were purchased from SILANTES (Germany). Mice were habituated for >4 days (usually 1 week) to the unlabeled l-^12^C_6_-lysine diet before being fed the l-^13^C_6_-lysine diet for 14 or 21 days. This labeling strategy has previously been thoroughly assessed and found to be safe, nontoxic, and without effect on development, growth, or behavior of mice that were kept for more than four generations on the SILAC diet (*21*). All mice whose food consumption variability across the whole labeling period was significantly higher than the one measured before acclimation were excluded from the study. Sample size was determined on the basis of previous statistical analyses of protein lifetimes (*18*). All animals were fed ad libitum, had unrestricted access to water, and were sacrificed at 21 months of age at the end of the labeling period. Food consumption and weight of the animals were monitored and recorded daily to eventually exclude mice that were not eating regularly (all mice whose average food consumption across the whole labeling period was >0.25 g with respect to their previous food consumption would have been excluded from the study).

### Brain tissue extraction and synaptic fractionation

Brain extraction, tissue dissection, and fraction purification were performed as previously described (*19*). Briefly, after dissection of brain cortex and cerebellum, the tissues from four mice were pooled to obtain sufficient material for subsequent fractionations. This pool is referred to as “biological replicate” in the text. Tissues were homogenized using a glass-Teflon homogenizer and underwent sequential step gradient centrifugation using Ficoll in sucrose buffer to obtain the P2′ fraction [see supplementary figure 21 from (*19*)]. Immediately after preparation, homogenates and synaptic fraction were snap-frozen in liquid nitrogen and stored at −80°C until further analysis.

### Mass spectrometry

Sample protein concentration was determined with a BCA kit (Thermo Fisher Scientific, USA). For each sample, 100 μg of protein was loaded on precast NuPAGE gels (Thermo Fisher Scientific, USA). Gels were run at constant voltage, stained overnight with Coomassie Blue, and destained with ultrapure double-distilled water. Next, each lane was cut into 23 gel pieces using an in-house–made gel cutter and processed for in-gel digestion using trypsin [for details, see (*18*)]. The eluted peptides were dried and resuspended in 12 fractions for LC-MS in an online UltiMate 3000 RSLCnano high-performance liquid chromatography (HPLC) system, coupled online to a Q-Exactive-HF. Peptides were desalted on a reversed-phase C18 precolumn, which was switched online after 3 min to the analytical column (30 cm; prepared in-house using ReproSil-Pur C18 AQ 1.9-μm reversed-phase resin). Peptides were separated over an 88-min gradient from 5 to 50% buffer B (80% acetonitrile and 0.1% formic acid). MS data were acquired by scanning precursors from 350 to 1600 Da at a resolution of 60,000 at mass/charge ratio (*m/z*) of 200. The top 30 precursor ions were chosen for MS2 by using data-dependent acquisition mode at a resolution of 15,000 at *m/z* of 200 with maximum injection time of 50 ms. For MS2, HCD (higher-energy C-trap dissociation) fragmentation was performed with the automatic gain control target fill value of 1 × 10^5^ ions. The precursors were isolated with a window of 1.4 Da. LC-MS acquisition setup and method were identical for both this aged cohort of mice and the previously published young adult cohort (*19*), and measurements of the two cohorts were performed on the same Q-Exactive-HF mass spectrometer within a few months.

### Mass spectrometry data analysis

The acquired “.raw” data and corresponding data from PXD010859 for the 5-month young adult mice were analyzed using MaxQuant version 1.6.17.0. The mouse UniProt database (downloaded August 2020) was used for identifying proteins. Label multiplicity was set to “2,” and ^13^C_6_-lysine was ticked as “heavy,” providing median heavy to light (H/L) values for each protein group that were further analyzed for determining protein turnover (see below). Note that the final MaxQuant output file (ProteinGroups_SF.xlsx) provides only H/L values for a subset of identified proteins, for which both the heavy and light forms are detected. All contaminants as well as reverse and only-identified-by-site proteins were removed from further analyses.

### Protein lifetime determination

In detail, the median H/L ratios among detected peptides (>3) were determined for each protein. Protein lifetimes were determined using the previously published available scripts (https://github.com/malevra/protein-turnover) (*18*), where several technical and biological replicates are used for lifetime determinations. The calculations took into consideration the relative enrichment of amino acids in the precursor pool, as addressed in our previous works (*18*, *19*). We consider the effect of lysine reuse on the precursor pool that then follows a double-exponential instead of a monoexponential [as assumed for example in (*75*)]. Hence, the lifetimes are intrinsically corrected for lysine reuse in our approach. The lysine pool parameters were as follows: *a* = 0.034277, *b* = 0.444865, and *r* = 11.836573. Before fitting, the data were inspected for consistency, and all cases where only one labeling time point was found for one protein group or the labeling was decreased during the pulse were not further considered for subsequent analyses. Variability in lifetime determination was defined as 95% confidence interval of the fitting for each protein group as previously described (*18*). For reliability of these measures and further explanations on the precision of protein lifetime determinations, refer to (*18*, *19*).

### Protein level determination

Protein quantification was based on “unique and razor peptide.” The options “Match Between Runs” and “Re-quantify” were turned on. Protein quantification was performed using the LFQ algorithm, with LFQ min ratio count set to “2.” LFQ values from both H and L measurements were summed to one value per protein group, median-normalized, and averaged for each tissue across technical and biological replicates as well as across SILAC labeling pulses.

### Data analysis, representation, and statistics

All analyses were performed with the help of Microsoft Excel, MATLAB 2017, Python, Perseus (v1.6.14.0), and GraphPad Prism (v8/9). Venn diagrams were initially generated with InteractiVenn (www.interactivenn.net/). To identify rLL or rSL proteins in the 21-month-old mice versus the 5-month-old mice (Figs. 2 to 4 and figs. S4 and S6 to S9), the lifetimes from the 21-month-old aged mice were linearly rescaled to the respective lifetimes of the 5-month-old tissues and fractions. The following rescaling factors, determined on the basis of the median of protein lifetimes, were used: 21.7% cortex homogenate, 24.6% cerebellum homogenate, 15.1% cortex synaptic fraction, and 18.1% cerebellum synaptic fraction. Relative lifetime differences were expressed as log_2_FC determined by the lifetime ratio of the 21 months scaled versus 5 months (log_2_FC). When averaging log_2_FC values across tissues and fractions for display purposes, only proteins with an increase or decrease in at least three of the four datasets were considered (including brain cortex, brain cerebellum, synaptic cortical fraction, and synaptic cerebellar fraction; for details, see table S3). For the coefficient of determination data matrix shown in Fig. 1C, only protein lifetimes found in all four groups were considered and *r*^2^ was calculated using Perseus 1.6.14.0. For Fig. 1D, only proteins measured in cortex homogenate whose lifetime confidence intervals did not exceed 100% of the lifetime values were used and grouped following organelle and/or functional affiliation as described below. *P* values were calculated with GraphPad Prism and as specified in the respective figures. For the one-way analysis of variance (ANOVA), if applicable, we used Brown-Forsythe and Welch tests. Multiple comparison correction was performed with Tukey posttest in the case of ordinary ANOVA and Dunnett (*n* < 50) or Games-Howell (*n* > 50) for Brown-Forsythe and Welch ANOVA.

### Bioinformatics and determination of protein features

Proteins in Fig. 1D were categorized in accordance to their organelle and/or functional affiliation using our previously manually curated lists (*19*), as also detailed in table S1. For the category-based representation of lifetimes in Fig. 1D, only proteins with the upper bound of the confidence intervals not exceeding two times the respective lifetime were considered. The lists of proteins connected to NDDs (Fig. 1, E to G) were manually curated using UniProt (*76*) and STRING v11 (*77*). Cortex and cerebellum homogenates and their average relative lifetime changes were assigned a specific cell type (fig. S7E) using the data provided by (*39*). Proteins were only considered specific for a cell type if their annotated expression surpassed the expression in the other three cell types by log_2_FC > 1. Thus, 640 proteins were assigned a unique cell type, and protein lifetime changes between young adult and aged brains could be compared between cell subpopulations. For the calculation of the Pearson’s correlations to the protein features (Fig. 3, A to C), a custom Python script was used to retrieve the amino acid sequences and calculate length and averaged amino acid compositions from UniProt identifiers. Glycosylation site information was retrieved from UniProt. Isoelectric point and GRAVY score were calculated using the ProtParam module in Biopython (https://biopython.org). The disordered fraction (IUPL) was obtained from IUPred (*78*), and the intrinsically disordered score was retrieved from MobiDB (*79*). For each protein sequence, the average biosynthetic cost (avECPA) was calculated using the values per amino acid previously described (*50*). Single amino acids were further classified as “metabolically expensive,” when their metric for energy cost, normalized by amino acid decay rate, is >60. Reversely, amino acids were classified as “metabolically affordable” when their metric is <20. Metrics are based on the calculations for humans from (*50*).

### Gene ontology, pathways, and functional enrichment analysis

Functional enrichment analyses were performed either with STRING v11 (*77*), with the WEB-based Gene SeT AnaLysis Toolkit 2019 (*80*), or with SynGOs (*24*). The STRING networks in fig. S6 were generated by setting the minimum required interaction score to 0.7 (high confidence), hiding disconnected nodes, and performing *k*-means clustering with three clusters. For the functional enrichment, we relied on both overrepresentation enrichment analysis (ORA; Fig. 2) and gene set enrichment analysis (GSEA; Fig. 3). All figures were assembled using Adobe Illustrator (CS6 or 2021).

## Supplementary Materials

### This PDF file includes:

Figs. S1 to S10 Text S1 and S2 References

## Notes

View/request a protocol for this paper from *Bio-protocol*.
