# Supplementary material for: Protein lifetimes in aged brains reveal a proteostatic adaptation linking physiological aging to neurodegeneration
Source: Sci Adv. 2022 May 20;8(20):eabn4437. doi: 10.1126/sciadv.abn4437 (PMC9122331; doi:10.1126/sciadv.abn4437)
Supplement: Supplementary file 1 — Figs. S1 to S10 Text S1 and S2 References [file sciadv.abn4437_sm.pdf]

Science Advances
AAAS

Supplementary Materials for

Protein lifetimes in aged brains reveal a proteostatic adaptation linking physiological aging to neurodegeneration

Verena Kluever, Belisa Russo, Sunit Mandad, Nisha Hemandhar Kumar, Mihai Alevra, Alessandro Ori, Silvio O. Rizzoli, Henning Urlaub, Anja Schneider*, Eugenio F. Fornasiero*

*Corresponding author. Email: anja.schneider@dzne.de (A.S.); efornas@gwdg.de (E.F.F.)

Published 20 May 2022, *Sci. Adv.* **8**, eabn4437 (2022)
DOI: 10.1126/sciadv.abn4437

The PDF file includes:

Figs. S1 to S10
Legends for tables S1 to S7
Text S1 and S2
References

Other Supplementary Material in this manuscript includes the following:

Tables S1 to S7

Supplementary texts

Text S1: Interpretation of concomitant lifetime and protein abundance changes (related to Fig. S8B).

Text S2: Changes in protein lifetimes with regional or synaptic specificities that could be explained by biochemical properties (related to Fig. S10).

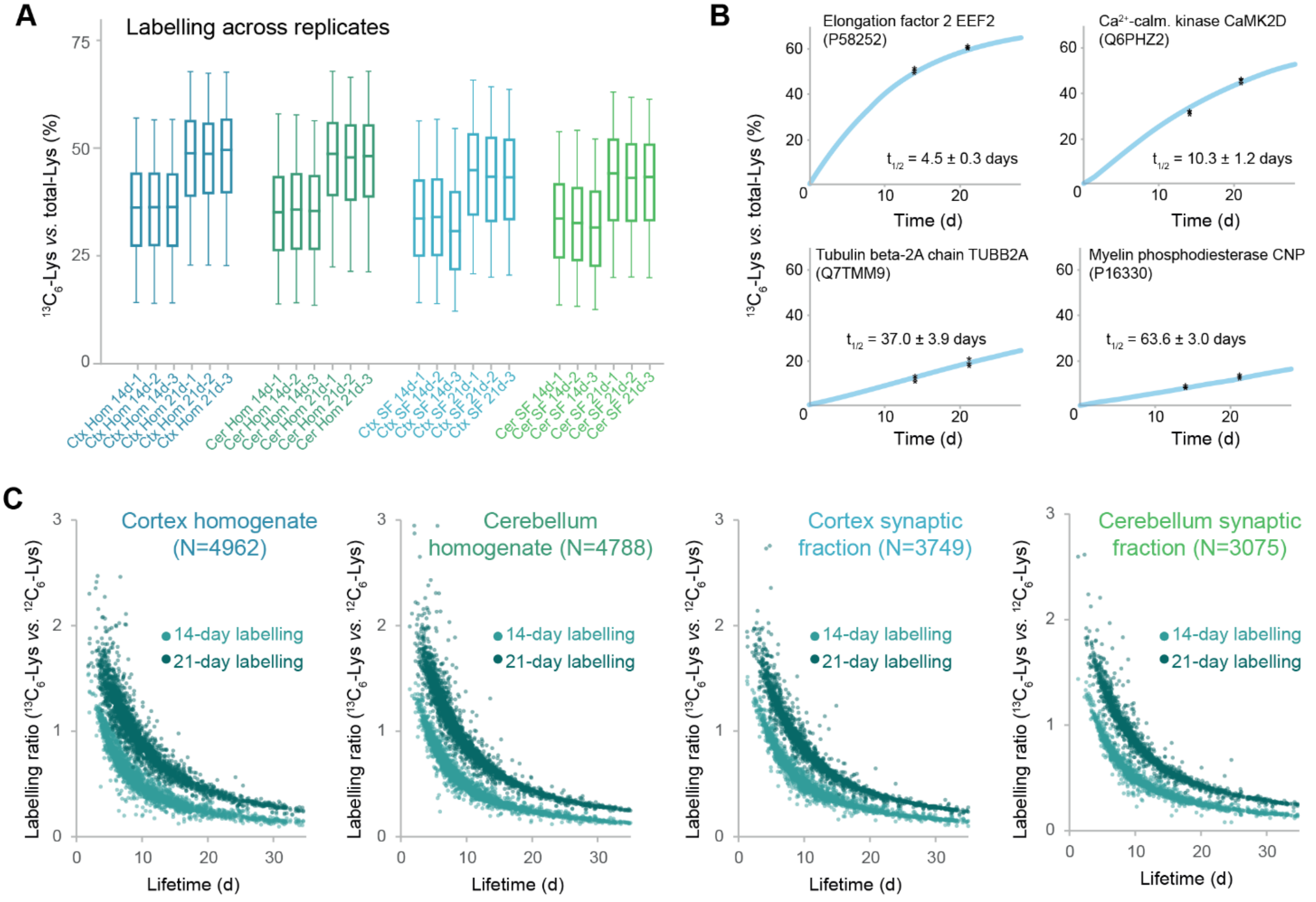

D

	Peptide number	Protein number	Protein lifetimes	Average lifetime (d)	Median lifetime (d)
Ctx Hom	74,921	4,962	3,252	11.41	8.82
Cer Hom	75,455	4,788	2,692	11.92	9.38
Ctx SF	56,377	3,749	2,431	13.70	10.88
Cer SF	38,571	3,075	1,880	14.35	11.22

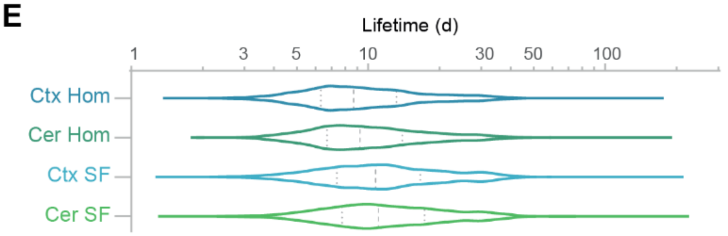

Fig. S1. Summary of labeling efficiencies, and overall data quality control for the metabolic labeling and the mass spectrometry measurements in the aged mouse brain cortex, cerebellum, and respective synaptic fractions. (**A**) Boxplots summarizing the percentage of $^{13}C_6$-lysin (heavy) versus total lysin for each time point and for each single biological replicate for cerebellum total homogenate, cortex total homogenate and the respective synaptic fractions. Boxplots represent median, 25th to 75th percentile with whiskers showing 5th-95th percentile. (**B**) Representative fittings for four proteins of the cortex homogenate with different lifetimes, calculated as in (*18*). Note that for several biological replicates (black asterisks) the variation is virtually nonexistent, mirroring the reproducibility of the data. (**C**) Scatter plots, obtained after fitting as described in (*18*), summarizing the interdependence of labeling ratio and lifetimes. The different color hues indicate different labeling times. (**D**) Supplementary Table summarizing the number of proteins, peptides, lifetimes, and relative average lifetime and median for the four datasets. **e,** Violin plots representing the distribution of lifetimes calculated in days. The thicker segmented line in the middle of each plot represents the median, while the thinner segmented lines represent lower 25th percentile and higher 75th percentile. Note that the graph has a logarithmic scale.

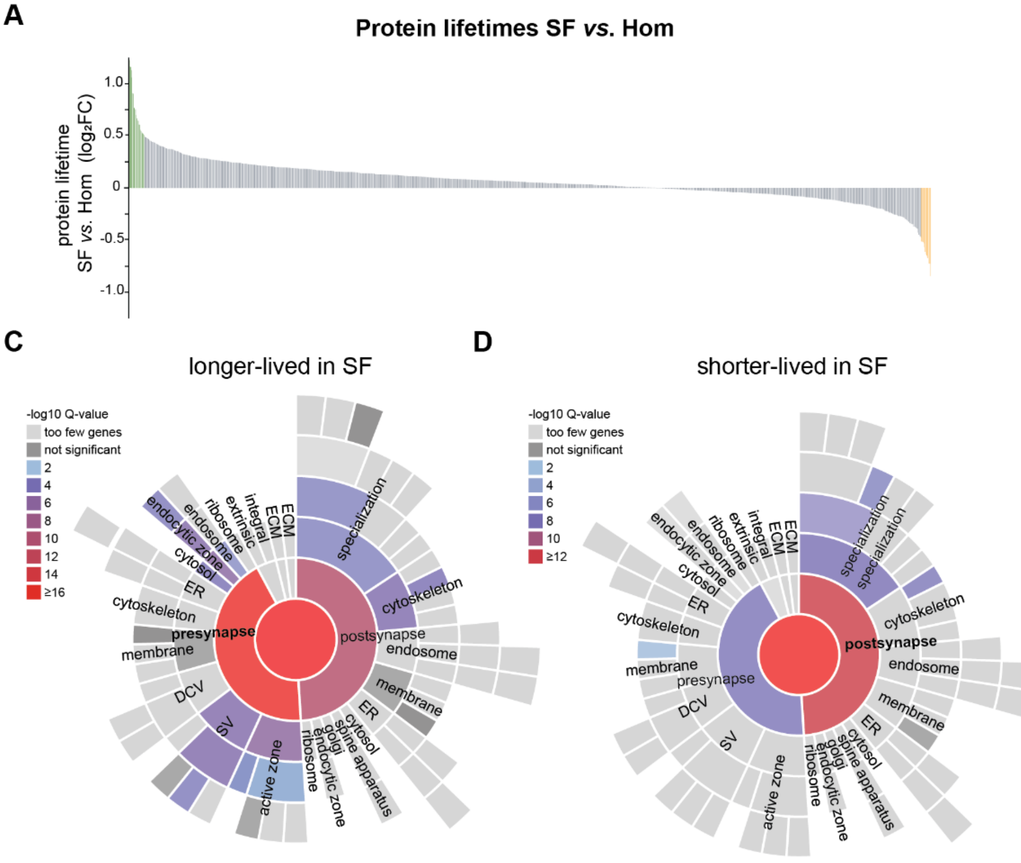

B

Protein ID	Gene name	SF *vs.* Hom (log_2FC)
P17809	Slc2a1	1.22
P24288	Bcat1	1.16
Q9QXL2	Kif21a	1.16
Q9Z0S1	Bpnt1	1.13
Q9D0E1	Hnrnpm	1.06
Q9CZD3	Gars1	0.91
Q148V7	Relch	0.90
Q9Z268	Rasal1	0.77
P68510	Ywhah	0.76
Q9CPV4	Glod4	0.75
Q5SSL4	Abr	0.70
Q8VCT3	Rnpep	0.67
Q69ZK0	Prex1	0.64
Q9JMC3	Dnaja4	0.64
Q91Z53	Grhpr	0.61
Q62095	Ddx3y	0.60
Q9DBC7	Prkar1a	0.56
Q99MN1	Kars1	0.54
G5E895	Akr1b10	0.53
Q8R127	Sccpdh	0.52
Q99LX0	Park7	0.52
O88531	Ppt1	0.51
P04370	Mbp	-0.51
Q9JIW9	Ralb	-0.51
P32233	Drg1	-0.52
P01831	Thy1	-0.53
O35963	Rab33b	-0.57
Q93092	Taldo1	-0.61
O70172	Pip4k2a	-0.63
P60202	Plp1	-0.65
P08551	Nefl	-0.66
Q9QUR8	Sema7a	-0.67
P68181	Prkacb	-0.72
Q5EBJ4	Ermn	-0.72
B9EKR1	Ptprz1	-0.73
Q9JL62	Gltp	-0.84

Fig. S2. Analysis of proteins longer- or shorter-lived in the synaptic fraction compared to the respective homogenates. We calculated the ratios of protein lifetimes in the synaptic fraction *vs.* its respective homogenate for both cortex and cerebellum. For 1143 proteins, ratios from both brain regions were obtained and averaged. Log_2FCs of these averages are shown as bars in (**A**). The color-coded bars represent all proteins with a $log_2FC > 0.5$ (green) or < -0.5 (yellow), which are also detailed in (**B**). All values can be inspected in **table S1**. For synaptic gene ontology enrichment analysis, SynGO (*24*) was used, inputting the proteins with $log_2FC > 0.15$ or < -0.15 for longer-lived (**C**) and shorter-lived (**D**) proteins in synaptic fraction *vs.* homogenate, respectively.

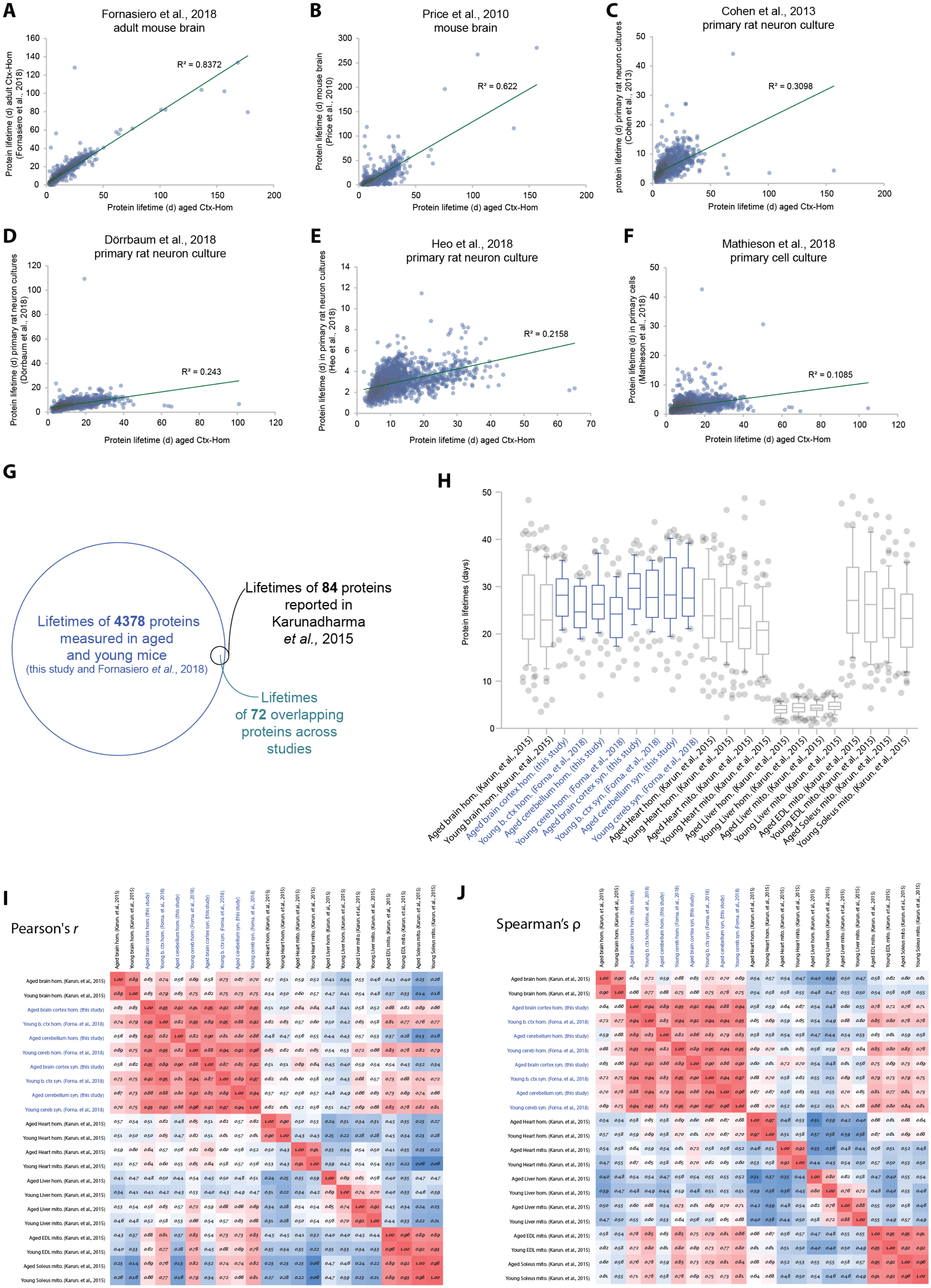

Fig. S3. Comparison of protein lifetimes between our aged cortex homogenate dataset and previously-published datasets. (**A, B**) Lifetimes acquired from in vivo experiments in the mouse brain; our own previously generated dataset

from young adult mice (*19*) and data produced by (*20*). (**C-F**) Protein lifetimes from in vitro data are generally less correlated, as previously described (*19*). Data were retrieved from (*23*, *81–83*). Scatterplot representations include the squared Pearson's *r*. (**G**) Venn diagrams of lifetimes measured in this study together with the previously published data from our group (*19*) compared to Karunadharma *et al.*, 2015 (*6*), showing that 72 proteins are common to all studies. Although two completely different labelling technologies and mass spectrometry analysis workflows were used, the overlap between the mitochondrial proteins of the two studies is sufficient to carry on subsequent analyses. (**H**) Protein lifetime distributions across the different studies, for both aged and young tissues. Our lifetime results are perfectly in line with those previously reported by (*6*), since the overall average lifetimes of these proteins in the brain in both studies are in the range of ~25-30 days. This is quite remarkable, especially since we used two different labels ($^{13}C_6$-lysine while they used $^{2}H_3$-leucine), confirming the validity of both studies. Some small differences that might be due to the fact that we used different brain regions, different MS methods, different protein extraction methods, and different mouse ages. The main finding of the work of Rabinovitch and colleagues is entirely confirmed by our data. This finding can be resumed as follows: once the lifetimes are normalized to the mean of the respective tissue and treatment group, the relative lifetime differences across mitochondrial proteins are conserved. As an example, some components of Complex I, such as Ndufs 1, Ndufs 2, Ndufs 3, Ndufs 4, Ndufs 6, Ndufs 7, Ndufa 4, Ndufa 6 and Ndufa 7, tend to have relatively shorter lifetimes than the mean of the respective tissue and treatment group for these mitochondrial proteins. Likewise, most of Complex V proteins tend to have relatively longer lifetimes. For the complete set, refer to the rightmost part of the **table S2**, showing the normalized values. (**I, J**) The similarities across studies summarized by Pearson's r and Spearman's ρ of the data. The average Pearson's r and Spearman's ρ of our lifetimes vs. the brain fractions of both young and aged mice are 0.68 ± 0.02 and 0.70 ± 0.01 (p-values <0.0001), indicating good agreement. Interestingly enough, the lifetimes from our study have even better correlations with the other tissues reported in Karunadharma *et al.*, 2015 than their own brain samples (our samples vs. their other tissues Pearson's *r* 0.59 ± 0.01 and Spearman's ρ 0.65 ± 0.01; while their brain samples vs. their other tissues Pearson's *r* 0.43 ± 0.03 and Spearman's ρ 0.53 ± 0.01). This indicates that the two studies are in very good agreement. Using the original data from Karunadharma *et al.*, 2015, also reported in **table S2**, we also compared the normal brain *vs.* the young brain and confirmed that also in their findings there is a significant lengthening of protein lifetimes in the aged brain (~7% higher in the aged brain for this selection of mitochondrial proteins, paired T-test p = 0.00025). As for the extent of this difference, we found a very similar value for this subset of proteins (averaging 7.13% when we consider all brain regions and subcellular fractions, but still significant with a paired T-test p < 0.0001). We also tested, using the same group– and treatment-wise normalization used in Karunadharma *et al.*, 2015, whether the lifetimes of these proteins correlate to any parameters in our bioinformatic collection. The only parameter with a significant correlation is the beta-sheet content of these proteins (*r* = 0.38, *P* = 0.006). This result is in line with the main finding of Karunadharma *et al.*, 2015, since it is likely that proteins with more compact structures (hence with higher beta-sheet content), are more stable.

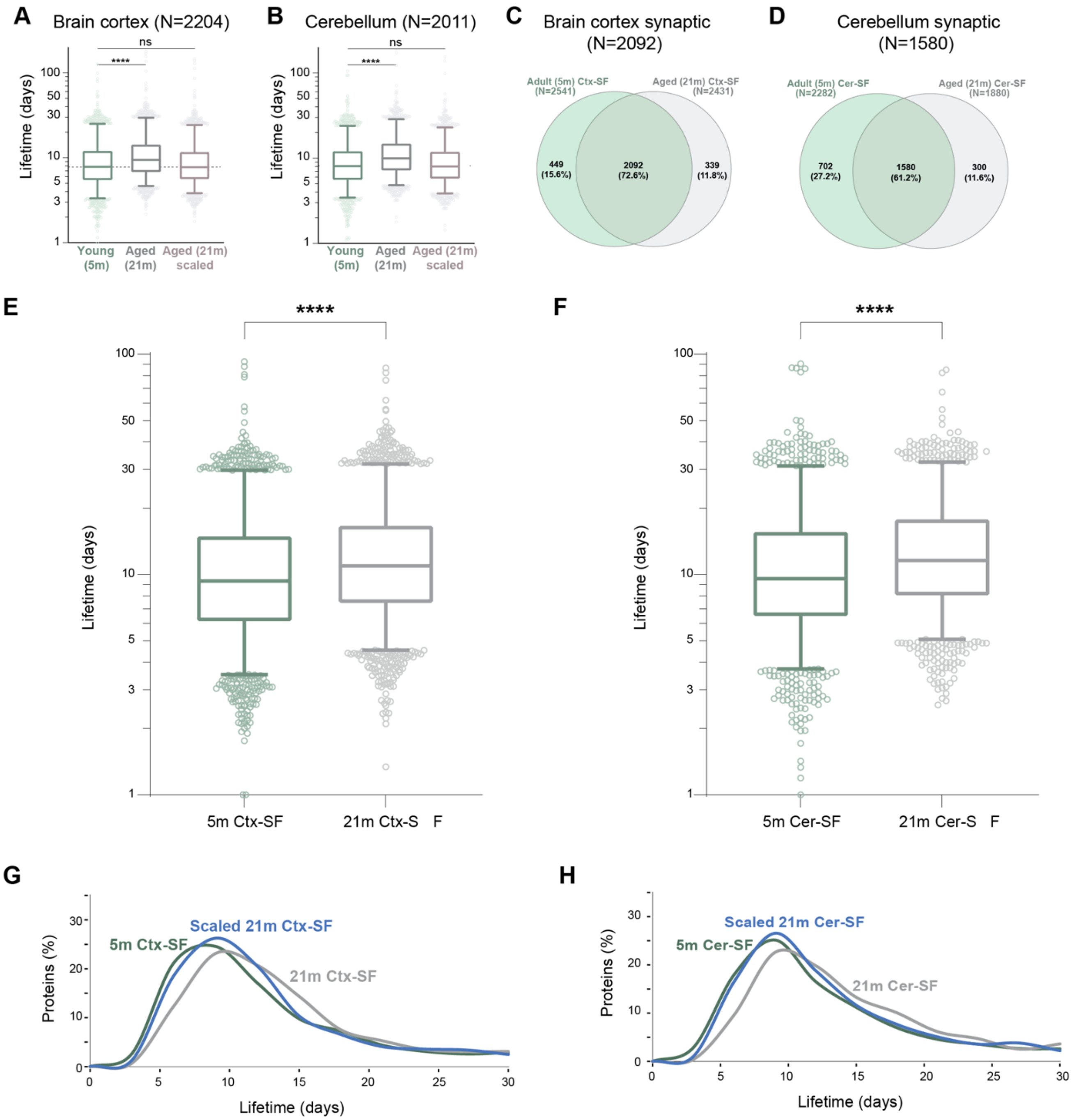

Fig. S4. Brain protein lifetime changes in synaptic fractions, before and after median rescaling (supplementing main **Fig. 2**). (**A**) Comparison of protein lifetimes in the cortex of 5m and 21m old mice. Turnover of the aged brain cortex is on average 21.7% slower compared to young adult. After median linear rescaling, accounting for this difference, lifetimes are not significantly different (nonparametric Wilcoxon matched-pairs signed rank test, N=2204, *P*-value: **** ≤ 0.0001; boxplots represent median, 25th to 75th percentile with whiskers showing 5th-95th percentile). (**B**) The turnover of the aged cerebellum proteome is also slower compared to the young adult cerebellum (24.3% slower at 21m *vs.* 5m). After median linear scaling, protein lifetimes show no significant difference (Wilcoxon matched-pairs signed rank test, N=2011, *P*-value: **** ≤ 0.0001). (**C, D**) Venn diagrams of lifetimes measured in this study ('aged mice'; 21m) and previously published data from our group (young adult mice'; 5m; (*19*) in synaptic fractions. For details on how synaptic fractions were prepared please refer to the methods and to our previous work (*19*). (**E, F**) Comparison of protein lifetimes in the cortex of 5m and 21m old mice. The turnover of the aged brain cortex synaptic fraction is on average slower compared to the young adult (15.14% in the SF cortex and 18.10% in the SF cerebellum). Boxplots represent median, 25th to 75th percentile with whiskers showing 5th-95th percentile. (**G, H**) Continuous distributions of lifetimes, showing the effect of median linear rescaling for the synaptic fractions, similar to what is represented in panels **A** and **B**.

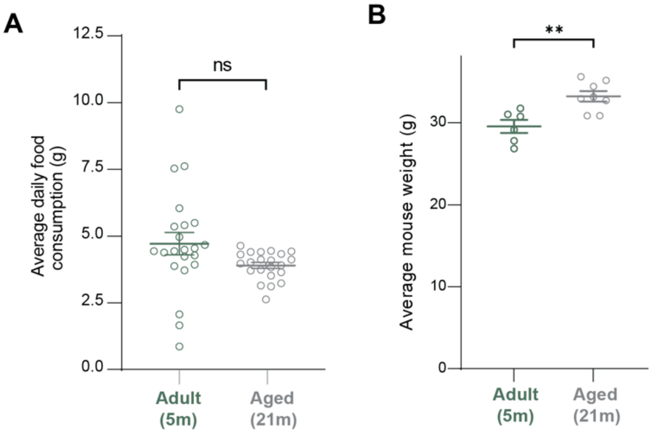

Fig. S5. Analysis of food consumption in mice at 5 and 21 months of age. (**A**) Average food consumption per day, measured in grams, indicating that young adult and aged animals consume on average the same amount of food (~4 grams per day). (**B**) average mouse weight in grams. Mean ± SEM, two-sided t-test *P*-value ** ≤ 0.01. For these measurements, a total amount of 14 mice was used.

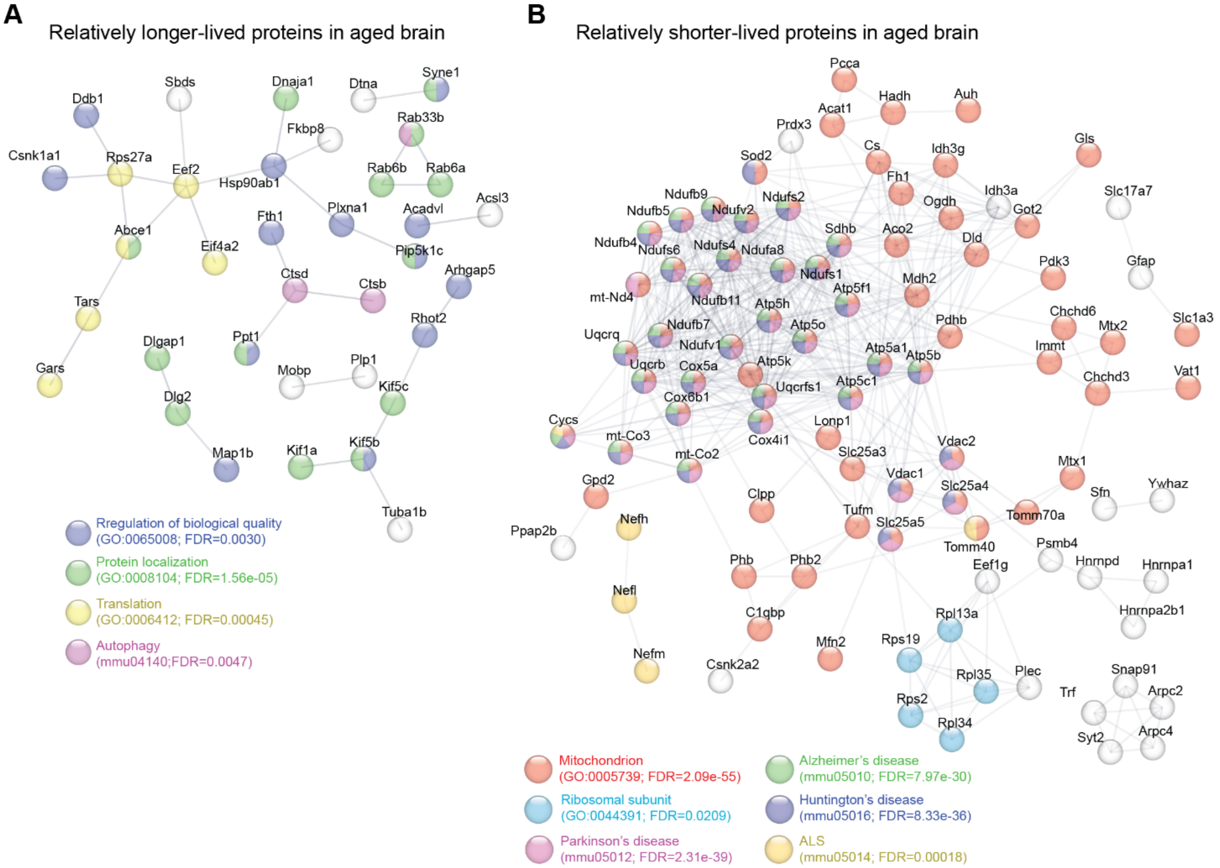

Fig. S6. STRING representation of the relatively-longer- and relatively-shorter-lived proteins in the aged brain. (**A**) rLL proteins in the aged brain (top 10% of consistently longer-lived proteins in all 4 datasets, resulting in 72 proteins) were analyzed with STRING v11 (*28*) and three representative GOs and one pathway are color coded. For the complete analysis results see **table S3**. Note that among these proteins there are several implicated in quality control, protein localization and also some key players in autophagy, such as Cathepsin C (an abundant lysosomal cysteine proteinase) and Cathepsin B (also known as APP secretase). (**B**) same as '**A**' for rSL proteins in the aged brain (128 final number of proteins analyzed, with results reported in **table S3**). As also explained in the main text, several among these proteins are mitochondrial components implicated in mitochondrial function and metabolism, with a direct link to NDDs, as also highlighted by the very highly significant false discovery rates (FDRs) for Alzheimer's, Parkinson's, and Huntington's.

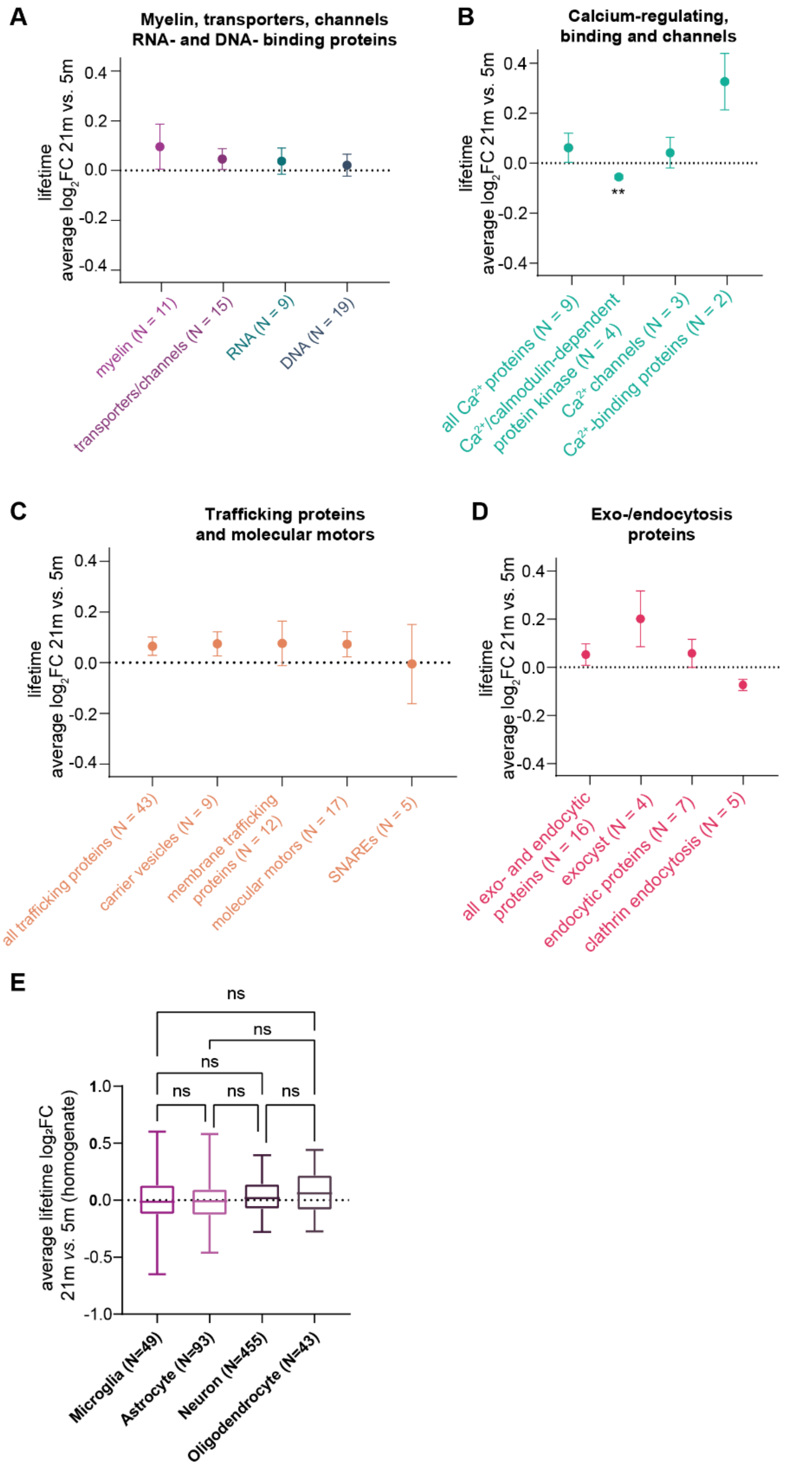

Fig. S7. Remaining categories for protein lifetimes changes supplementing Fig. 2I. (**A-D**) Proteins either rLL or rSL in aged mice according to their manually-curated organelle and/or functional affiliation (see (*19*) and **table S5**). The mean ± SEM of the $\log_2$FC (21m *vs*. 5m) is represented. Numbers of proteins per category are indicated in parentheses. Significance against the list of consistently changing lifetimes was calculated using Brown-Forsythe and Welch analysis of variance (ANOVA) followed by Dunnett multiple comparison correction (*P*-values: ** ≤ 0.01 and not significant if nothing is indicated). (**E**) There are no significant differences in changes in lifetimes between microglia, astrocytes, neurons, and oligodendrocytes (assigned according to (48), see Methods for details). The average log2FC 21m vs. 5m of cortex and cerebellum homogenate was calculated and proteins were sorted according to their dominant cell type. Boxplots represent median, 25th to 75th percentile with whiskers showing 5th-95th percentile; p>0.05 for all comparisons, one-way ANOVA with Tukey's multiple comparison correction.

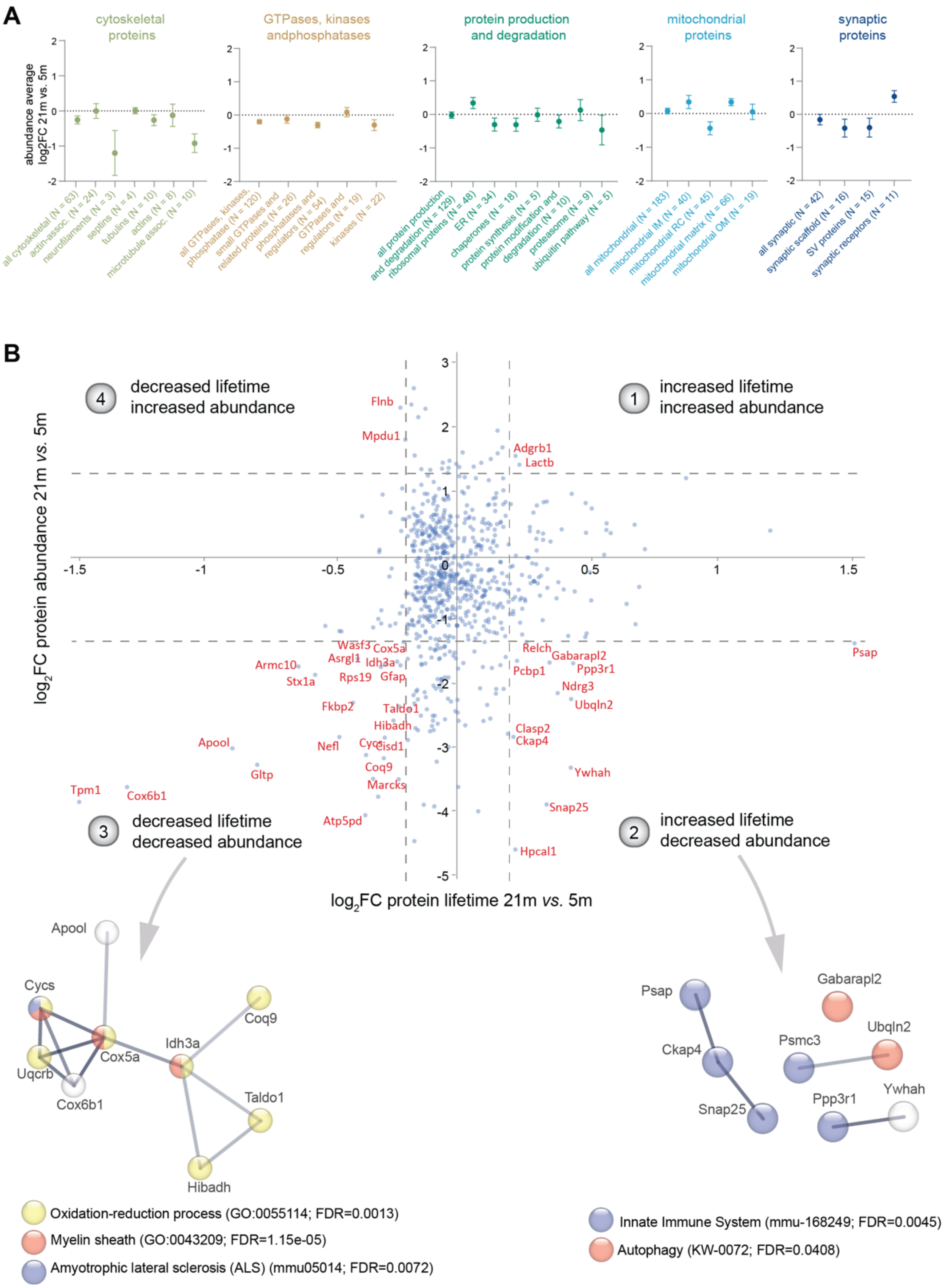

Fig. S8. Correlation between protein lifetime changes and protein abundance changes. (**A**) Protein abundance changes corresponding to the classes of protein lifetimes presented in main **Fig. 2I**. Protein abundances, calculated as LFQ and detailed in **tables S1** and **S5**, are summarized for the respective affiliations, following the categorization used in main **Fig. 2I**. The mean ± SEM of the log_2FC LFQ (21m *vs*. 5m) is represented. Numbers of proteins per category are indicated in parentheses. Significance against the list of consistently changing abundances across tissue groups was calculated using Brown-Forsythe and Welch analysis of variance (ANOVA) followed by Dunnett multiple comparison correction. No significant differences were found. (**B**) Log_2 fold changes of protein abundance and lifetimes of aged 21m mice and young adult 5m mice are plotted (751 proteins consistently changed (>3) in both lifetime and abundance change, see **table S5**) and the top 10% in each quadrant are highlighted in red. Insets below show the STRING analysis of quadrants 2 and 3. See **Supplementary Text 1** for interpretation

A

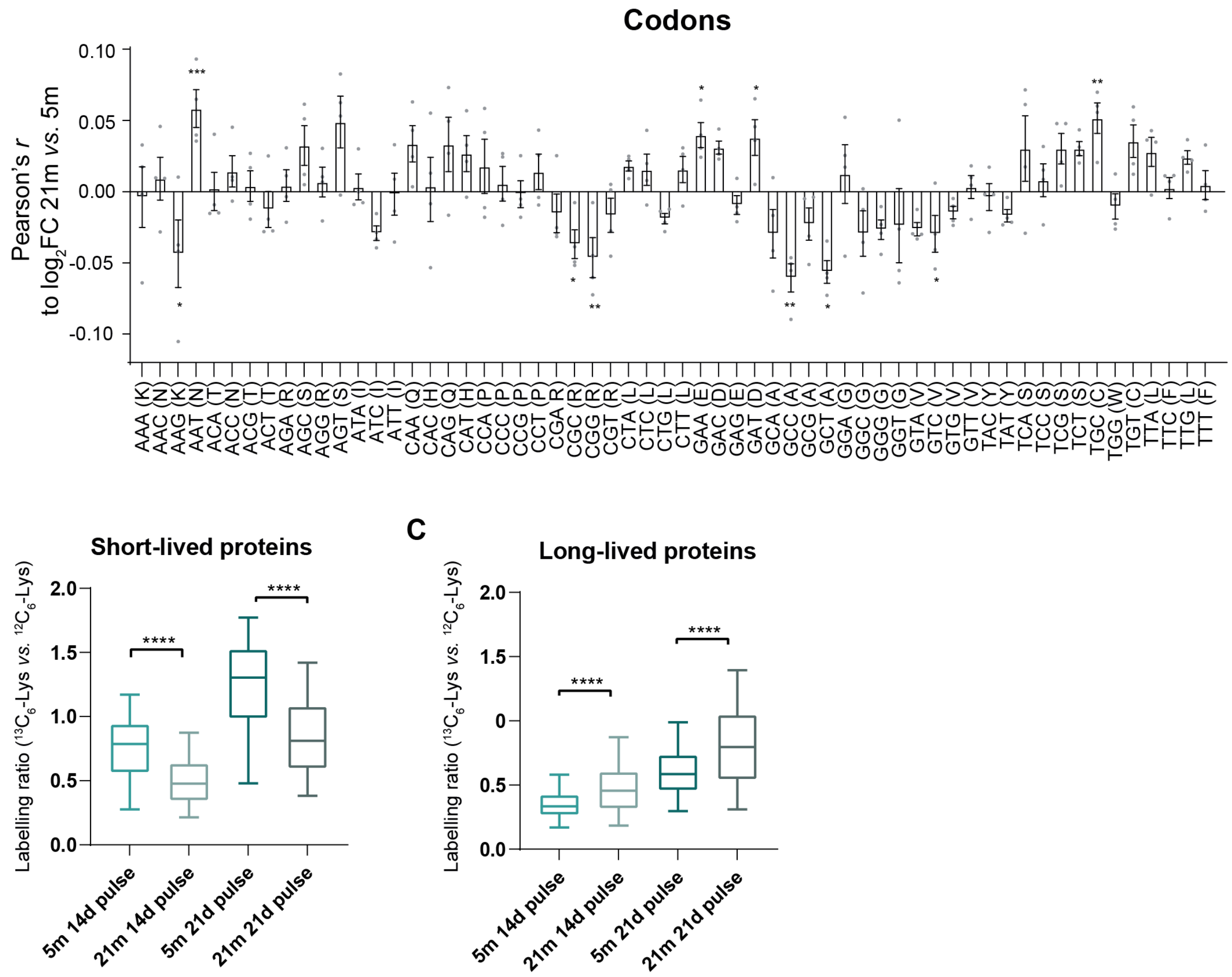

Fig. S9. Correlation to protein lifetime change during brain aging to amino acid composition, codon composition and relative labeling in short-lived and long-lived proteins. (**A**) Pearson's coefficients show correlations of lifetime $\log_2$ fold changes to codon usage. Bars represent mean ± SEM and points are changes in the individual datasets (Pearson's *P*-values: * ≤ 0.05, ** ≤ 0.01, *** ≤0.001). (**B, C**) Compression of aged proteome lifetimes as seen in **Fig. 3F** verified with the original data of heavy to light lysine ratios (ratio H/L) to exclude observations due to our workflow and lifetime fitting (see **table S7**). In **B,** short-lived proteins (<25th percentile of all lifetimes measured in this study, averaged between 5m and 21m mice) are depicted and significantly lower H/L ratios are observed in the 21m aged mice (grey shaded colors), meaning slower turnover of these proteins. This is true for both the 14d as well as the 21d pulse duration. (**C**) same as in **B**, but for generally long-lived proteins (>75th percentile). Paired two-sided t-tests between 5m and 21m, *P*-values **** ≤0.0001; boxplots represent median, 25th to 75th percentile with whiskers showing 5th-95th percentile.

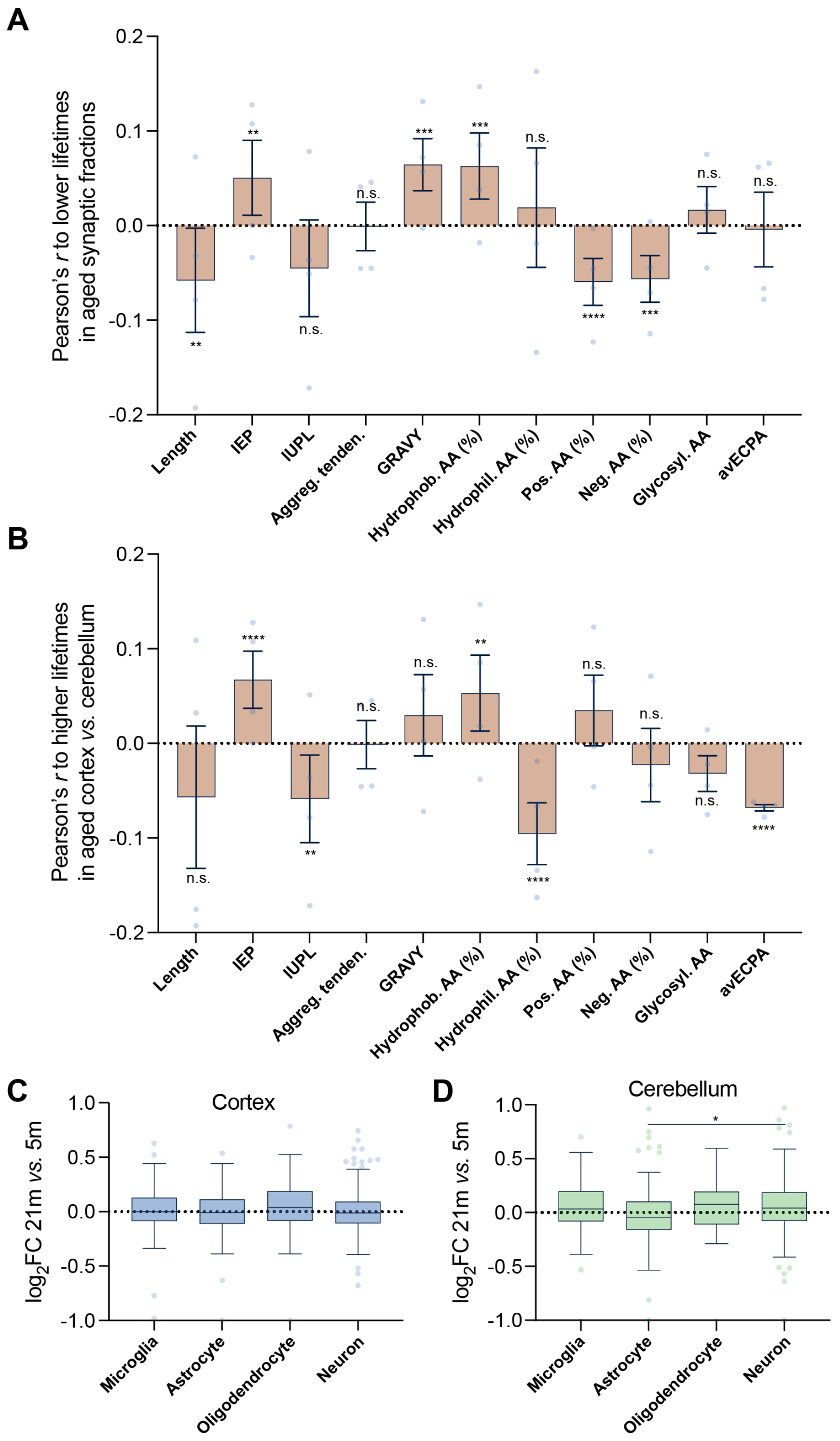

Fig. S10. Correlation between biochemical parameters in synaptic fractions, brain cortex and cerebellum. Lifetime shifts in different cell types. (**A, B**) Pearson's *r* correlation analysis between the biochemical parameters also used in **Fig. 4** and the relative lifetime changes observed in 21m mice vs. the 5m in synaptic fractions or in cortex and cerebellum. Correlations are shown either to lower lifetimes in synaptic fractions (**A**) or higher lifetimes in the brain cortex (**B**). Linear correlation significances are tested including all lifetime changes found at least three times in all fractions (**table S6**). (**C, D**) Distributions of log_2FC indicating the relative change in protein lifetimes in 21m *vs.* 5m mice across different cellular populations as previously defined (*40*) and summarized in **table S6**. All significances were tested with ANOVA followed by Tukey post-test normalized by multiple comparison.

Supplementary Text 1 (related to Fig. S8B)

In general, we observed a significant positive correlation between changes in protein levels and changes in protein turnover during aging (Pearson's $r = 0.0536$, $P = 0.0042$). Nevertheless, since turnover measurements capture the instantaneous velocity of protein replacement, it is not surprising that this correlation is small. Ideally, the more appropriate comparison between turnover and protein levels should involve one protein turnover measurement and a set of two protein level measurements, the first performed shortly before and the second performed shortly after the turnover measurement. When measured in this fashion, the relative protein level changes and the respective protein turnover measurement would correlate well. As an example, a protein whose lifetime increases will tend to be accumulated in a short temporal scale. In the particular case of our work, in which we compare two turnover measurements (aged adult and young adult), we would need to involve four protein level measurements (before and after the turnover measurements in both age groups).

In reality, protein abundance measurements are known to suffer from reproducibility issues (*84*), and such comparisons may hold small correlations across time spans, since protein levels can change with complex trajectories over time. This implies that protein turnover measurements and protein levels should be considered as two inherently different measurements, similarly to mRNA turnover and mRNA level measurements (*85*). Thus, the two measurements should be considered complementary rather than consequential, which explains why it has always been difficult to interpret the comparison between protein lifetime changes and protein level changes (*85*).

Nonetheless, we were interested in identifying the proteins whose lifetimes consistently change in these long time-scales, either in the same or in the opposite direction with respect to protein levels, which enables us, in some instances, to infer changes in protein production and degradation trajectories. For this purpose, we compared the lifetime changes (log_2FC protein lifetime 21m *vs.* 5m) with their change in protein abundance in aged animals vs. adult animals (log_2FC protein abundance 21m *vs.* 5m). We only considered proteins with up- or downregulation in at least three out of the four dataset (including brain cortex, brain cerebellum, synaptic cortical fraction and synaptic cerebellar fraction) comparisons for both abundance and lifetime, which resulted in 751 proteins (**table S5** and **fig. S8B**). These proteins can be subdivided following four scenarios: 1) Proteins with increased lifetime and increased abundance (which are less efficiently degraded and thus tend to get accumulated); 2) Proteins with increased lifetimes but decreased abundance (which have slow translation rates and tend to become less abundant); 3) Proteins with decreased lifetimes and decreased abundance (which are more efficiently degraded and, as a consequence, have decreased levels); 4) Proteins with decreased lifetimes but increased abundance (which have high translation rates and tend to get accumulated). This analysis revealed that among these four classes, the most populated is the third scenario (39.15% of all 751 proteins considered), where proteins have decreased levels due to increased degradation (**fig. S8B**, lower left corner).

To restrict our analysis to the most relevant variations among these 751 proteins, we considered proteins whose changes were higher than defined thresholds (see **fig. S8B** for details). We thus considered more in detail 39 proteins (in red in **fig. S8B**). Also among these selected proteins, the most populated scenario is scenario 3 (decreased lifetimes and decreased abundance). These include specifically some mitochondrial proteins, such as the Cytochrome C protein CYCS and the Cytochrome C Oxidase Subunit 5A, in line with increased degradation of mitochondrial proteins in the aged brain (*86*). Interestingly, some of these encompass myelin components, probably reflecting unproductive myelination, as observed in the optic nerve of aged animals (*87*). Among proteins with a putative slower translation efficiency (second scenario), we have observed some proteins with a role in autophagy such

as Ubiquilin 2, which regulates protein degradation mechanisms and pathways including the ubiquitin-proteasome system (**fig. S8B**, lower right corner).

Other degradative pathways, including the lysosomal pathway, show paradoxical increased protein expression, but actual decline in function in the aged brain and other organs (*35*, *55*, *88*). We also followed up on chaperone-mediated autophagy, which was recently connected to neuronal proteostasis in aging and NDD (*7*). In this context, Hsc70 recognizes the 'KFERQ' motifs of proteins, facilitating their lysosomal degradation. While overall lifetime changes are significantly positively correlated to the presence of canonical KFERQ motifs (Pearson's $r = 0.045$, P = 0.0164), this correlation is not significant once corrected for protein length, which in itself is positively correlated to KFERQ motif presence (Pearson's $r = -0.007$, P=0.6872) (*89*). In other words, it seems that there is no preferential role of lysosomal degradation, but rather it suggests that longer proteins in general are more sensitive to aging-mediated derangement. For the moment, until more direct measures of *in vivo* protein production and degradation become available, this type of considerations require careful examination. As discussed above, the whole trajectory, the 'history' of protein production and degradation might lead to misleading interpretations of the data. As an additional consideration, we have analyzed here the measurements that are globally changed in all our four datasets (cortex, cerebellum and two respective synaptic fractions), but we cannot exclude that tissue- or fraction differences exist, as also summarized in the main text.

Supplementary Text 2 (related to Fig. S10)

To test whether changes in protein lifetimes that have regional or synaptic specificities could be explained by biochemical properties, we correlated the proteins lifetime changes to the parameters shown in Fig. 4. Significant correlations were found when considering synaptic fractions vs. the total brain homogenate and also when comparing two different brain regions (**Fig. S10A-B**).

For synapses, the hydrophobicity and the GRAVY hydropathic score are significantly correlated to lower lifetimes in aged animals (**Fig. S10A**), which is also corroborated by a negative correlation with negatively- and positively-charged amino acids. The most likely explanation for these findings is that many membrane proteins (enriched in hydrophobic residues) may be affected in ageing, possibly through a specific effect on synaptic transmission (*90*) that might prelude neurodegenerative alterations, in line with changes in synaptic vesicle protein lifetimes in APP mice (*71*).

For the differences between cortex and cerebellum, we noticed two main trends. First, there is a negative correlation between preferential lifetime changes in the cortex of aged mice with hydrophilic amino acid percentages, which might indicate that there is a different protein solubility tolerance between these two brain regions (*4*). Second, we observed a negative correlation to the content of expensive amino acids, also suggestive of differences in the metabolism of these two brain regions in aged animals.

We also addressed if in brain cortex and in cerebellum of aged mice there are cell-specific shifts in protein lifetimes (**Fig. S10C-D**). This analysis was based on proteins that can be affiliated specifically to different cell lineages in the brain, based on previous measurements (*40*), and also summarized in **table S6**. While in the cortex we found no statistical difference between cell types (**Fig. S10C**), in the cerebellum we found that there is a significant difference in the lifetime shift between neurons and astrocytes (**Fig. S10D**; ANOVA multiple comparisons, followed by Tukey post-test adjusted for multiple comparisons). The finding is intriguing but remains for the moment correlative until more direct forms of validations of lifetime changes in these cells will be available.

Supplementary Table captions (note that these files are available as separate files).

Table S1: In *vivo* pulsing of 21-month aged mice with $^{13}C_6$ Lysine and LC-MS protein measurements of brain cortex, cerebellum, and their respective synaptic fractions. Subsequent protein lifetime determination using the previously established method described in (*18*, *19*). In this table we summarize identified peptides, protein groups, LFQ values, H/L ratios ($^{13}C_6$-Lys *vs.* $^{12}C_6$), lifetimes in days and their confidence intervals, as well as the categorization used for **Fig. 1** and **fig. S1**. Lifetime differences between synaptic fraction and homogenate are also detailed here.

Table S2: Comparison to a previous work (*6*) reporting lifetimes from brain mitochondrial proteins in young and aged animals.

Table S3: Comparison of protein lifetimes in 21m aged and 5m young adult mice. Here we summarize the lifetimes in young and aged mice, the linear scaling and the consistently changed lifetimes. It also contains the full heatmap and GO analysis for **Fig. 2** and **fig. S4, S6**.

Table S4: rLL proteins in the aged mouse brain are connected to neurodegenerative disease (NDD). Details and references for proteins linked to NDDs that are marked with a red box in **Fig. 2E** are listed here.

Table S5: Log_2 fold changes of protein lifetimes (and LFQ abundance) in 21m aged *vs*. 5m young adult mice, categorized as in Fig. 1 and in Fornasiero et al, 2018. Used in **Fig. 2I** and **fig. S7, S8**.

Table S6: Differences in relative lifetime changes (log_2FC 21m *vs*. 5m) between datasets (cortex - cerebellum and homogenate - synaptic fraction). Here we summarized row z-score normalized lifetime changes for the heatmaps and for the full GO analysis represented in **Fig. 3**. Additionally, the cell type assignments are reported in accordance to previously-published results (*40*), used in **fig. S7.**

Table S7: Correlations between biochemical protein parameters and turnover. In this table we summarize the annotated protein features and their Pearson correlation to lifetime changes in the aged brain. We also list the relatively shorter-, middle- and longer-lived proteins with their features as well as the original H/L data used in **Fig. 4** and **fig. S9**.

REFERENCES AND NOTES

1. Y. Hou, X. Dan, M. Babbar, Y. Wei, S. G. Hasselbalch, D. L. Croteau, V. A. Bohr, Ageing as a risk factor for neurodegenerative disease. *Nat. Rev. Neurol.* **15**, 565–581 (2019).

2. D. M. Walther, M. Mann, Accurate quantification of more than 4000 mouse tissue proteins reveals minimal proteome changes during aging. *Mol. Cell. Proteomics* **10**, (2011).

3. A. Ori, B. H. Toyama, M. S. Harris, T. Bock, M. Iskar, P. Bork, N. T. Ingolia, M. W. Hetzer, M. Beck, Integrated transcriptome and proteome analyses reveal organ-specific proteome deterioration in old rats. *Cell Syst.* **1**, 224–237 (2015).

4. E. Kelmer Sacramento, J. M. Kirkpatrick, M. Mazzetto, M. Baumgart, A. Bartolome, S. Di Sanzo, C. Caterino, M. Sanguanini, N. Papaevgeniou, M. Lefaki, D. Childs, S. Bagnoli, E. Terzibasi Tozzini, D. Di Fraia, N. Romanov, P. H. Sudmant, W. Huber, N. Chondrogianni, M. Vendruscolo, A. Cellerino, A. Ori, Reduced proteasome activity in the aging brain results in ribosome stoichiometry loss and aggregation. *Mol. Syst. Biol.* **16**, e9596 (2020).

5. W. F. Ward, The relentless effects of the aging process on protein turnover. *Biogerontology* **1**, 195–199 (2000).

6. P. P. Karunadharma, N. Basisty, Y. A. Chiao, D. F. Dai, R. Drake, N. Levy, W. J. Koh, M. J. Emond, S. Kruse, D. Marcinek, M. J. Maccoss, P. S. Rabinovitch, Respiratory chain protein turnover rates in mice are highly heterogeneous but strikingly conserved across tissues, ages, and treatments. *FASEB J.* **29**, 3582–3592 (2015).

7. M. Bourdenx, A. Martín-Segura, A. Scrivo, J. A. Rodriguez-Navarro, S. Kaushik, I. Tasset, A. Diaz, N. J. Storm, Q. Xin, Y. R. Juste, E. Stevenson, E. Luengo, C. C. Clement, S. J. Choi, N. J. Krogan, E. V Mosharov, L. Santambrogio, F. Grueninger, L. Collin, D. L. Swaney, D. Sulzer, E. Gavathiotis, A. M. Cuervo, Chaperone-mediated autophagy prevents collapse of the neuronal metastable proteome. *Cell* **184**, 2696–2714.e25 (2021).

8. C. López-Otín, M. A. Blasco, L. Partridge, M. Serrano, G. Kroemer, The hallmarks of aging. *Cell* **153**, 1194–1217 (2013).

9. C. Hetz, Adapting the proteostasis capacity to sustain brain healthspan. *Cell* **184**, 1545–1560 (2021).

10. D.-F. Dai, P. P. Karunadharma, Y. A. Chiao, N. Basisty, D. Crispin, E. J. Hsieh, T. Chen, H. Gu, D. Djukovic, D. Raftery, R. P. Beyer, M. J. MacCoss, P. S. Rabinovitch, Altered proteome turnover and remodeling by short-term caloric restriction or rapamycin rejuvenate the aging heart. *Aging Cell* **13**, 529–539 (2014).

11. S. E. Kruse, P. P. Karunadharma, N. Basisty, R. Johnson, R. P. Beyer, M. J. MacCoss, P. S. Rabinovitch, D. J. Marcinek, Age modifies respiratory complex I and protein homeostasis in a muscle type-specific manner. *Aging Cell* **15**, 89–99 (2016).

12. P. P. Karunadharma, N. Basisty, D.-F. Dai, Y. A. Chiao, E. K. Quarles, E. J. Hsieh, D. Crispin, J. H. Bielas, N. G. Ericson, R. P. Beyer, V. L. MacKay, M. J. MacCoss, P. S.

Rabinovitch, Subacute calorie restriction and rapamycin discordantly alter mouse liver proteome homeostasis and reverse aging effects. *Aging Cell* **14**, 547–557 (2015).

13. N. Basisty, J. G. Meyer, B. Schilling, Protein turnover in aging and longevity. *Proteomics* **18**, e1700108 (2018).

14. E. S. Vincow, R. E. Thomas, G. E. Merrihew, M. J. MacCoss, L. J. Pallanck, Slowed protein turnover in aging Drosophila reflects a shift in cellular priorities. *J. Gerontol. A Biol. Sci. Med. Sci.* **76**, 1734–1739 (2021).

15. M. Visscher, S. De Henau, M. H. E. Wildschut, R. M. van Es, I. Dhondt, H. Michels, P. Kemmeren, E. A. Nollen, B. P. Braeckman, B. M. T. Burgering, H. R. Vos, T. B. Dansen, Proteome-wide changes in protein turnover rates in C. elegans models of longevity and age-related disease. *Cell Rep.* **16**, 3041–3051 (2016).

6. L. Wang, S. S. Davis, M. Borch Jensen, I. A. Rodriguez-Fernandez, C. Apaydin, G. Juhasz, B. W. Gibson, B. Schilling, A. Ramanathan, S. Ghaemmaghami, H. Jasper, JNK modifies neuronal metabolism to promote proteostasis and longevity. *Aging Cell* **18**, e12849 (2019).

17. N. Basisty, A. Holtz, B. Schilling, Accumulation of “Old Proteins” and the critical need for MS-based protein turnover measurements in aging and longevity. *Proteomics* **20**, e1800403 (2020).

18. M. Alevra, S. Mandad, T. Ischebeck, H. Urlaub, S. O. Rizzoli, E. F. Fornasiero, A mass spectrometry workflow for measuring protein turnover rates in vivo. *Nat. Protoc.* **14**, 3333–3365 (2019).

19. E. F. Fornasiero, S. Mandad, H. Wildhagen, M. Alevra, B. Rammner, S. Keihani, F. Opazo, I. Urban, T. Ischebeck, M. S. Sakib, M. K. Fard, K. Kirli, T. P. Centeno, R. O. Vidal, R.-U. Rahman, E. Benito, A. Fischer, S. Dennerlein, P. Rehling, I. Feussner, S. Bonn, M. Simons, H. Urlaub, S. O. Rizzoli, Precisely measured protein lifetimes in the mouse brain reveal differences across tissues and subcellular fractions. *Nat. Commun.* **9**, 4230 (2018).

20. J. C. Price, S. Guan, A. Burlingame, S. B. Prusiner, S. Ghaemmaghami, Analysis of proteome dynamics in the mouse brain. *Proc. Natl. Acad. Sci. U.S.A.* **107**, 14508–14513 (2010).

21. M. Krüger, M. Moser, S. Ussar, I. Thievessen, C. A. Luber, F. Forner, S. Schmidt, S. Zanivan, R. Fässler, M. Mann, SILAC mouse for quantitative proteomics uncovers kindlin-3 as an essential factor for red blood cell function. *Cell* **134**, 353–364 (2008).

22. K. Klann, G. Tascher, C. Münch, Functional translatome proteomics reveal converging and dose-dependent regulation by mTORC1 and eIF2α. *Mol. Cell* **77**, 913–925.e4 (2020).

23. S. Heo, G. H. Diering, C. H. Na, R. S. Nirujogi, J. L. Bachman, A. Pandey, R. L. Huganir, Identification of long-lived synaptic proteins by proteomic analysis of synaptosome protein turnover. *Proc. Natl. Acad. Sci. U.S.A.*, 201720956 (2018).

24. F. Koopmans, P. van Nierop, M. Andres-Alonso, A. Byrnes, T. Cijsouw, M. P. Coba, L. N. Cornelisse, R. J. Farrell, H. L. Goldschmidt, D. P. Howrigan, N. K. Hussain, C. Imig, A. P. H. de

Jong, H. Jung, M. Kohansalnodehi, B. Kramarz, N. Lipstein, R. C. Lovering, H. MacGillavry, V. Mariano, H. Mi, M. Ninov, D. Osumi-Sutherland, R. Pielot, K. H. Smalla, H. Tang, K. Tashman, R. F. G. Toonen, C. Verpelli, R. Reig-Viader, K. Watanabe, J. van Weering, T. Achsel, G. Ashrafi, N. Asi, T. C. Brown, P. De Camilli, M. Feuermann, R. E. Foulger, P. Gaudet, A. Joglekar, A. Kanellopoulos, R. Malenka, R. A. Nicoll, C. Pulido, J. de Juan-Sanz, M. Sheng, T. C. Südhof, H. U. Tilgner, C. Bagni, À. Bayés, T. Biederer, N. Brose, J. J. E. Chua, D. C. Dieterich, E. D. Gundelfinger, C. Hoogenraad, R. L. Huganir, R. Jahn, P. S. Kaeser, E. Kim, M. R. Kreutz, P. S. McPherson, B. M. Neale, V. O'Connor, D. Posthuma, T. A. Ryan, C. Sala, G. Feng, S. E. Hyman, P. D. Thomas, A. B. Smit, M. Verhage, SynGO: An evidence-based, expert-curated knowledge base for the synapse. *Neuron* **103**, 217–234.e4 (2019).

25. B. H. Toyama, J. N. Savas, S. K. Park, M. S. Harris, N. T. Ingolia, J. R. Yates, M. W. Hetzer, Identification of long-lived proteins reveals exceptional stability of essential cellular structures. *Cell* **154**, 971–982 (2013).

26. E. Lau, Q. Cao, D. C. M. Ng, B. J. Bleakley, T. U. Dincer, B. M. Bot, D. Wang, D. A. Liem, M. P. Y. Lam, J. Ge, P. Ping, A large dataset of protein dynamics in the mammalian heart proteome. *Sci. Data* **3**, 160015 (2016).

27. L. F. Lacey, O. N. Keene, J. F. Pritchard, A. Bye, Common noncompartmental pharmacokinetic variables: Are they normally or log-normally distributed? *J. Biopharm. Stat.* **7**, 171–178 (1997).

28. C. Sato, N. R. Barthélemy, K. G. Mawuenyega, B. W. Patterson, B. A. Gordon, J. Jockel-Balsarotti, M. Sullivan, M. J. Crisp, T. Kasten, K. M. Kirmess, N. M. Kanaan, K. E. Yarasheski, A. Baker-Nigh, T. L. S. Benzinger, T. M. Miller, C. M. Karch, R. J. Bateman, Tau kinetics in neurons and the human central nervous system. *Neuron* **97**, 1284–1298.e7 (2018).

29. M. Allen, M. M. Carrasquillo, C. Funk, B. D. Heavner, F. Zou, C. S. Younkin, J. D. Burgess, H.-S. Chai, J. Crook, J. A. Eddy, H. Li, B. Logsdon, M. A. Peters, K. K. Dang, X. Wang, D. Serie, C. Wang, T. Nguyen, S. Lincoln, K. Malphrus, G. Bisceglio, M. Li, T. E. Golde, L. M. Mangravite, Y. Asmann, N. D. Price, R. C. Petersen, N. R. Graff-Radford, D. W. Dickson, S. G. Younkin, N. Ertekin-Taner, Human whole genome genotype and transcriptome data for Alzheimer's and other neurodegenerative diseases. *Sci. Data* **3**, 160089 (2016).

30. K. W. Li, A. B. Ganz, A. B. Smit, Proteomics of neurodegenerative diseases: Analysis of human post-mortem brain. *J. Neurochem.* **151**, 435–445 (2019).

31. D.-H. Cho, T. Nakamura, S. A. Lipton, Mitochondrial dynamics in cell death and neurodegeneration. *Cell. Mol. Life Sci.* **67**, 3435–3447 (2010).

32. D. J. R. Lane, S. Ayton, A. I. Bush, Iron and Alzheimer's disease: An update on emerging mechanisms. *J. Alzheimers Dis.* **64**, S379–S395 (2018).

33. A. Weiland, Y. Wang, W. Wu, X. Lan, X. Han, Q. Li, J. Wang, Ferroptosis and its role in diverse brain diseases. *Mol. Neurobiol.* **56**, 4880–4893 (2019).

34. S. Kaushik, A. M. Cuervo, Proteostasis and aging. *Nat. Med.* **21**, 1406–1415 (2015).

35. A. K. Pollard, E. L. Craig, L. Chakrabarti, Mitochondrial complex 1 activity measured by spectrophotometry is reduced across all brain regions in ageing and more specifically in neurodegeneration. *PLOS ONE* **11**, e0157405 (2016).

36. K. L. Stauch, P. R. Purnell, L. M. Villeneuve, H. S. Fox, Proteomic analysis and functional characterization of mouse brain mitochondria during aging reveal alterations in energy metabolism. *Proteomics* **15**, 1574–1586 (2015).

37. N. Almanzar, J. Antony, A. S. Baghel, I. Bakerman, I. Bansal, B. A. Barres, P. A. Beachy, D. Berdnik, B. Bilen, D. Brownfield, C. Cain, C. K. F. Chan, M. B. Chen, M. F. Clarke, S. D. Conley, S. Darmanis, A. Demers, K. Demir, A. de Morree, T. Divita, H. du Bois, H. Ebadi, F. H. Espinoza, M. Fish, Q. Gan, B. M. George, A. Gillich, R. Gòmez-Sjöberg, F. Green, G. Genetiano, X. Gu, G. S. Gulati, O. Hahn, M. S. Haney, Y. Hang, L. Harris, M. He, S. Hosseinzadeh, A. Huang, K. C. Huang, T. Iram, T. Isobe, F. Ives, R. C. Jones, K. S. Kao, J. Karkanias, G. Karnam, A. Keller, A. M. Kershner, N. Khoury, S. K. Kim, B. M. Kiss, W. Kong, M. A. Krasnow, M. E. Kumar, C. S. Kuo, J. Lam, D. P. Lee, S. E. Lee, B. Lehallier, O. Leventhal, G. Li, Q. Li, L. Liu, A. Lo, W.-J. Lu, M. F. Lugo-Fagundo, A. Manjunath, A. P. May, A. Maynard, A. McGeever, M. McKay, M. W. McNerney, B. Merrill, R. J. Metzger, M. Mignardi, D. Min, A. N. Nabhan, N. F. Neff, K. M. Ng, P. K. Nguyen, J. Noh, R. Nusse, R. Pálovics, R. Patkar, W. C. Peng, L. Penland, A. O. Pisco, K. Pollard, R. Puccinelli, Z. Qi, S. R. Quake, T. A. Rando, E. J. Rulifson, N. Schaum, J. M. Segal, S. S. Sikandar, R. Sinha, R. V Sit, J. Sonnenburg, D. Staehli, K. Szade, M. Tan, W. Tan, C. Tato, K. Tellez, L. B. T. Dulgeroff, K. J. Travaglini, C. Tropini, M. Tsui, L. Waldburger, B. M. Wang, L. J. van Weele, K. Weinberg, I. L. Weissman, M. N. Wosczyna, S. M. Wu, T. Wyss-Coray, J. Xiang, S. Xue, K. A. Yamauchi, A. C. Yang, L. P. Yerra, J. Youngyunpipatkul, B. Yu, F. Zanini, M. E. Zardeneta, A. Zee, C. Zhao, F. Zhang, H. Zhang, M. J. Zhang, L. Zhou, J. Zou; Tabula Muris Consortium, A single-cell transcriptomic atlas characterizes ageing tissues in the mouse. *Nature* **583**, 590–595 (2020).

38. V. Kluever, E. F. Fornasiero, Principles of brain aging: Status and challenges of modeling human molecular changes in mice. *Ageing Res. Rev.* **72**, 101465 (2021).

39. K. Sharma, S. Schmitt, C. G. Bergner, S. Tyanova, N. Kannaiyan, N. Manrique-Hoyos, K. Kongi, L. Cantuti, U.-K. Hanisch, M.-A. Philips, M. J. Rossner, M. Mann, M. Simons, Cell type– and brain region–resolved mouse brain proteome. *Nat. Neurosci.* **18**, 1819–1831 (2015).

40. M. M. Boisvert, G. A. Erikson, M. N. Shokhirev, N. J. Allen, The aging astrocyte transcriptome from multiple regions of the mouse brain. *Cell Rep.* **22**, 269–285 (2018).

41. K. J. Liang, E. S. Carlson, Resistance, vulnerability and resilience: A review of the cognitive cerebellum in aging and neurodegenerative diseases. *Neurobiol. Learn. Mem.* **170**, 106981 (2020).

42. A. Kurotani, A. A. Tokmakov, K.-I. Sato, V. E. Stefanov, Y. Yamada, T. Sakurai, Localization-specific distributions of protein pI in human proteome are governed by local pH and membrane charge. *BMC Mol. Cell Biol.* **20**, 36 (2019).

43. J. F. Dice, A. L. Goldberg, Relationship between in vivo degradative rates and isoelectric points of proteins. *Proc. Natl. Acad. Sci. U.S.A.* **72**, 3893–3897 (1975).

44. J. Krištić, F. Vučković, C. Menni, L. Klarić, T. Keser, I. Beceheli, M. Pučić-Baković, M. Novokmet, M. Mangino, K. Thaqi, P. Rudan, N. Novokmet, J. Sarac, S. Missoni, I. Kolčić, O. Polašek, I. Rudan, H. Campbell, C. Hayward, Y. Aulchenko, A. Valdes, J. F. Wilson, O. Gornik, D. Primorac, V. Zoldoš, T. Spector, G. Lauc, Glycans are a novel biomarker of chronological and biological ages. *J. Gerontol. A Biol. Sci. Med. Sci.* **69**, 779–789 (2014).

45. M. Frenkel-Pinter, S. Stempler, S. Tal-Mazaki, Y. Losev, A. Singh-Anand, D. Escobar-Álvarez, J. Lezmy, E. Gazit, E. Ruppin, D. Segal, Altered protein glycosylation predicts Alzheimer's disease and modulates its pathology in disease model Drosophila. *Neurobiol. Aging* **56**, 159–171 (2017).

6. R. Raghunathan, N. K. Polinski, J. A. Klein, J. D. Hogan, C. Shao, K. Khatri, D. Leon, M. E. McComb, F. P. Manfredsson, C. E. Sortwell, J. Zaia, Glycomic and proteomic changes in aging brain nigrostriatal pathway. *Mol. Cell. Proteomics* **17**, 1778–1787 (2018).

47. J. Lee, S. Ha, M. Kim, S.-W. Kim, J. Yun, S. Ozcan, H. Hwang, I. J. Ji, D. Yin, M. J. Webster, C. Shannon Weickert, J.-H. Kim, J. S. Yoo, R. Grimm, S. Bahn, H.-S. Shin, H. J. An, Spatial and temporal diversity of glycome expression in mammalian brain. *Proc. Natl. Acad. Sci. U.S.A.* **117**, 28743–28753 (2020).

8. J. Ivanisevic, K. L. Stauch, M. Petrascheck, H. P. Benton, A. A. Epstein, M. Fang, S. Gorantla, M. Tran, L. Hoang, M. E. Kurczy, M. D. Boska, H. E. Gendelman, H. S. Fox, G. Siuzdak, Metabolic drift in the aging brain. *Aging* **8**, 1000–1020 (2016).

49. K. P. Kepp, A quantitative model of human neurodegenerative diseases involving protein aggregation. *Neurobiol. Aging* **80**, 46–55 (2019).

50. H. Zhang, Y. Wang, J. Li, H. Chen, X. He, H. Zhang, H. Liang, J. Lu, Biosynthetic energy cost for amino acids decreases in cancer evolution. *Nat. Commun.* **9**, 4124 (2018).

51. L. Agozzino, K. A. Dill, Protein evolution speed depends on its stability and abundance and on chaperone concentrations. *Proc. Natl. Acad. Sci. U.S.A.* **115**, 9092–9097 (2018).

52. J. H. Roberts, F. Liu, J. M. Karnuta, M. C. Fitzgerald, Discovery of age-related protein folding stability differences in the mouse brain proteome. *J. Proteome Res.* **15**, 4731–4741 (2016).

53. N. B. Basisty, Y. Liu, J. Reynolds, P. P. Karunadharma, D. F. Dai, J. Fredrickson, R. P. Beyer, M. J. Maccoss, P. S. Rabinovitch, Stable isotope labeling reveals novel insights into ubiquitin-mediated protein aggregation with age, calorie restriction, and rapamycin treatment. *J. Gerontol. Ser. A Biol. Sci. Med. Sci.* **73**, 561–570 (2018).

54. Q. Yu, H. Xiao, M. P. Jedrychowski, D. K. Schweppe, J. Navarrete-Perea, J. Knott, J. Rogers, E. T. Chouchani, S. P. Gygi, Sample multiplexing for targeted pathway proteomics in aging mice. *Proc. Natl. Acad. Sci. U.S.A.* **117**, 9723–9732 (2020).

55. M. P. Mattson, Cellular actions of beta-amyloid precursor protein and its soluble and fibrillogenic derivatives. *Physiol. Rev.* **77**, 1081–1132 (1997).

56. A. Knupp, S. Mishra, R. Martinez, S. A. Small, S. Jayadev, J. E. Y. Correspondence, Depletion of the AD risk gene SORL1 selectively impairs neuronal endosomal traffic independent of amyloidogenic APP processing. *Cell Rep.* **31**, 107719 (2020).

57. K. E. Grear, I. F. Ling, J. F. Simpson, J. L. Furman, C. R. Simmons, S. L. Peterson, F. A. Schmitt, W. R. Markesbery, Q. Liu, J. E. Crook, S. G. Younkin, G. Bu, S. Estus, Expression of SORL1 and a novel SORL1 splice variant in normal and Alzheimers disease brain. *Mol. Neurodegener.* **4**, 46 (2009).

58. M. Steuble, T. M. Diep, P. Schätzle, A. Ludwig, M. Tagaya, B. Kunz, P. Sonderegger, Calsyntenin-1 shelters APP from proteolytic processing during anterograde axonal transport. *Biol. Open* **1**, 761–774 (2012).

59. I. V. Kurochkin, Insulin-degrading enzyme: Embarking on amyloid destruction. *Trends Biochem. Sci.* **26**, 421–425 (2001).

60. V. Stoka, V. Turk, B. Turk, Lysosomal cathepsins and their regulation in aging and neurodegeneration. *Ageing Res. Rev.* **32**, 22–37 (2016).

61. R. Tian, A. Abarientos, J. Hong, S. H. Hashemi, R. Yan, N. Dräger, K. Leng, M. A. Nalls, A. B. Singleton, K. Xu, F. Faghri, M. Kampmann, Genome-wide CRISPRi/a screens in human neurons link lysosomal failure to ferroptosis. *Nat. Neurosci.* **24**, 1020–1034 (2021).

62. C. Mukherjee, T. Kling, B. Russo, K. Miebach, E. Kess, M. Schifferer, L. D. Pedro, U. Weikert, M. K. Fard, N. Kannaiyan, M. Rossner, M.-L. Aicher, S. Goebbels, K.-A. Nave, E.-M. Krämer-Albers, A. Schneider, M. Simons, Oligodendrocytes provide antioxidant defense function for neurons by secreting ferritin heavy chain. *Cell Metab.* **32**, 259–272.e10 (2020).

63. K. P. Koster, A. Yoshii, Depalmitoylation by palmitoyl-protein thioesterase 1 in neuronal health and degeneration. *Front. Synaptic Neurosci.* **11**, 25 (2019).

64. E. Bomba-Warczak, S. L. Edassery, T. J. Hark, J. N. Savas, Long-lived mitochondrial cristae proteins in mouse heart and brain. *J. Cell Biol.* **220**, e202005193 (2021).

65. P. González-Rodríguez, E. Zampese, K. A. Stout, J. N. Guzman, E. Ilijic, B. Yang, T. Tkatch, M. A. Stavarache, D. L. Wokosin, L. Gao, M. G. Kaplitt, J. López-Barneo, P. T. Schumacker, D. J. Surmeier, Disruption of mitochondrial complex I induces progressive parkinsonism. *Nature* **599**, 650–656 (2021).

66. H. Wang, K. K. Dey, P. C. Chen, Y. Li, M. Niu, J. H. Cho, X. Wang, B. Bai, Y. Jiao, S. R. Chepyala, V. Haroutunian, B. Zhang, T. G. Beach, J. Peng, Integrated analysis of ultra-deep proteomes in cortex, cerebrospinal fluid and serum reveals a mitochondrial signature in Alzheimer's disease. *Mol. Neurodegener.* **15**, 1–20 (2020).

67. R. Yousefi, K. Jevdokimenko, V. Kluever, D. Pacheu-Grau, E. F. Fornasiero, Influence of subcellular localization and functional state on protein turnover. *Cell* **10**, 1747 (2021).

68. C. T. Schanzenbächer, S. Sambandan, J. D. Langer, E. M. Schuman, Nascent proteome remodeling following homeostatic scaling at hippocampal synapses. *Neuron* **92**, 358–371 (2016).

69. C. Glock, A. Biever, G. Tushev, B. Nassim-Assir, A. Kao, I. Bartnik, S. Tom Dieck, E. M. Schuman, The translatome of neuronal cell bodies, dendrites, and axons. *Proc. Natl. Acad. Sci. U.S.A.* **118**, e2113929118 (2021).

70. T. J. Hark, N. R. Rao, C. Castillon, T. Basta, S. Smukowski, H. Bao, A. Upadhyay, E. Bomba-Warczak, T. Nomura, E. T. O'Toole, G. P. Morgan, L. Ali, T. Saito, C. Guillermier, T. C. Saido, M. L. Steinhauser, M. H. B. Stowell, E. R. Chapman, A. Contractor, J. N. Savas, Pulse-chase proteomics of the app knockin mouse models of Alzheimer's disease reveals that synaptic dysfunction originates in presynaptic terminals. *Cell Syst.* **12**, 141–158.e9 (2021).

71. H. Liu, R. G. Sadygov, J. R. Yates, A model for random sampling and estimation of relative protein abundance in shotgun proteomics. *Anal. Chem.* **76**, 4193–4201 (2004).

72. A. Breschi, T. R. Gingeras, R. Guigó, Comparative transcriptomics in human and mouse. *Nat. Rev. Genet.* **18**, 425–440 (2017).

73. À. Bayés, M. O. Collins, M. D. R. Croning, L. N. van de Lagemaat, J. S. Choudhary, S. G. N. Grant, Comparative study of human and mouse postsynaptic proteomes finds high compositional conservation and abundance differences for key synaptic proteins. *PLOS ONE* **7**, e46683 (2012).

74. K. Swovick, K. A. Welle, J. R. Hryhorenko, A. Seluanov, V. Gorbunova, S. Ghaemmaghami, Cross-species comparison of proteome turnover kinetics. *Mol. Cell. Proteomics* **17**, 580–591 (2018).

5. E. J. Hsieh, N. J. Shulman, D.-F. Dai, E. S. Vincow, P. P. Karunadharma, L. Pallanck, P. S. Rabinovitch, M. J. MacCoss, Topograph, a software platform for precursor enrichment corrected global protein turnover measurements. *Mol. Cell. Proteomics* **11**, 1468–1474 (2012).

76. A. Bateman, M.-J. Martin, S. Orchard, M. Magrane, R. Agivetova, S. Ahmad, E. Alpi, E. H. Bowler-Barnett, R. Britto, B. Bursteinas, H. Bye-A-Jee, R. Coetzee, A. Cukura, A. Da Silva, P. Denny, T. Dogan, T. Ebenezer, J. Fan, L. G. Castro, P. Garmiri, G. Georghiou, L. Gonzales, E. Hatton-Ellis, A. Hussein, A. Ignatchenko, G. Insana, R. Ishtiaq, P. Jokinen, V. Joshi, D. Jyothi, A. Lock, R. Lopez, A. Luciani, J. Luo, Y. Lussi, A. MacDougall, F. Madeira, M. Mahmoudy, M. Menchi, A. Mishra, K. Moulang, A. Nightingale, C. S. Oliveira, S. Pundir, G. Qi, S. Raj, D. Rice, M. R. Lopez, R. Saidi, J. Sampson, T. Sawford, E. Speretta, E. Turner, N. Tyagi, P. Vasudev, V. Volynkin, K. Warner, X. Watkins, R. Zaru, H. Zellner, A. Bridge, S. Poux, N. Redaschi, L. Aimo, G. Argoud-Puy, A. Auchincloss, K. Axelsen, P. Bansal, D. Baratin, M.-C. Blatter, J. Bolleman, E. Boutet, L. Breuza, C. Casals-Casas, E. de Castro, K. C. Echioukh, E. Coudert, B. Cuche, M. Doche, D. Dornevil, A. Estreicher, M. L. Famiglietti, M. Feuermann, E. Gasteiger, S. Gehant, V. Gerritsen, A. Gos, N. Gruaz-Gumowski, U. Hinz, C. Hulo, N. Hyka-Nouspikel, F. Jungo, G. Keller, A. Kerhornou, V. Lara, P. Le Mercier, D. Lieberherr, T. Lombardot, X. Martin, P. Masson, A. Morgat, T. B. Neto, S. Paesano, I. Pedruzzi, S. Pilbout, L. Pourcel, M. Pozzato, M. Pruess, C. Rivoire, C. Sigrist, K. Sonesson, A. Stutz, S. Sundaram, M.

Tognolli, L. Verbregue, C. H. Wu, C. N. Arighi, L. Arminski, C. Chen, Y. Chen, J. S. Garavelli, H. Huang, K. Laiho, P. McGarvey, D. A. Natale, K. Ross, C. R. Vinayaka, Q. Wang, Y. Wang, L.-S. Yeh, J. Zhang, P. Ruch, D. Teodoro, UniProt: The universal protein knowledgebase in 2021. *Nucleic Acids Res.* **49**, D480–D489 (2021).

77. D. Szklarczyk, A. L. Gable, D. Lyon, A. Junge, S. Wyder, J. Huerta-Cepas, M. Simonovic, N. T. Doncheva, J. H. Morris, P. Bork, L. J. Jensen, C. von Mering, STRING v11: Protein-protein association networks with increased coverage, supporting functional discovery in genome-wide experimental datasets. *Nucleic Acids Res.* **47**, D607–D613 (2019).

78. Z. Dosztányi, V. Csizmok, P. Tompa, I. Simon, IUPred: Web server for the prediction of intrinsically unstructured regions of proteins based on estimated energy content. *Bioinformatics* **21**, 3433–3434 (2005).

79. D. Piovesan, M. Necci, N. Escobedo, A. M. Monzon, A. Hatos, I. Mičetić, F. Quaglia, L. Paladin, P. Ramasamy, Z. Dosztányi, W. F. Vranken, N. E. Davey, G. Parisi, M. Fuxreiter, S. C. E. Tosatto, MobiDB: Intrinsically disordered proteins in 2021. *Nucleic Acids Res.* **49**, D361–D367 (2021).

80. Y. Liao, J. Wang, E. J. Jaehnig, Z. Shi, B. Zhang, WebGestalt 2019: Gene set analysis toolkit with revamped UIs and APIs. *Nucleic Acids Res.* **47**, W199–W205 (2019).

81. L. L. D. Cohen, R. Zuchman, O. Sorokina, A. Müller, D. C. Dieterich, J. D. Armstrong, T. Ziv, N. E. Ziv, Metabolic turnover of synaptic proteins: Kinetics, interdependencies and implications for synaptic maintenance. *PLOS ONE* **8**, e63191 (2013).

82. A. R. Dörrbaum, L. Kochen, J. D. Langer, E. M. Schuman, Local and global influences on protein turnover in neurons and glia. *eLife* **7**, 1–24 (2018).

83. T. Mathieson, H. Franken, J. Kosinski, N. Kurzawa, N. Zinn, G. Sweetman, D. Poeckel, V. S. Ratnu, M. Schramm, I. Becher, M. Steidel, K. M. Noh, G. Bergamini, M. Beck, M. Bantscheff, M. M. Savitski, Systematic analysis of protein turnover in primary cells. *Nat. Commun.* **9**, 1–10 (2018).

84. D. L. Tabb, L. Vega-Montoto, P. A. Rudnick, A. M. Variyath, A.-J. L. Ham, D. M. Bunk, L. E. Kilpatrick, D. D. Billheimer, R. K. Blackman, H. L. Cardasis, S. A. Carr, K. R. Clauser, J. D. Jaffe, K. A. Kowalski, T. A. Neubert, F. E. Regnier, B. Schilling, T. J. Tegeler, M. Wang, P. Wang, J. R. Whiteaker, L. J. Zimmerman, S. J. Fisher, B. W. Gibson, C. R. Kinsinger, M. Mesri, H. Rodriguez, S. E. Stein, P. Tempst, A. G. Paulovich, D. C. Liebler, C. Spiegelman, Repeatability and reproducibility in proteomic identifications by liquid chromatography-tandem mass spectrometry. *J. Proteome Res.* **9**, 761–776 (2010).

85. A. B. Ross, J. D. Langer, M. Jovanovic, Proteome turnover in the spotlight: Approaches, applications, and perspectives. *Mol. Cell. Proteomics* **20**, 100016 (2021).

86. A. Popa-Wagner, R. E. Sandu, C. Cristin, A. Uzoni, K. A. Welle, J. R. Hryhorenko, S. Ghaemmaghami, Increased degradation rates in the components of the mitochondrial oxidative phosphorylation chain in the cerebellum of old mice. *Front. Aging Neurosci.* **10**, 1–10 (2018).

87. H. Attia, M. Taha, A. Abdellatif, Effects of aging on the myelination of the optic nerve in rats. *Int. J. Neurosci.* **129**, 320–324 (2019).

88. A. Cellerino, A. Ori, What have we learned on aging from omics studies? *Semin. Cell Dev. Biol.* **70**, 177–189 (2017).

89. P. Kirchner, M. Bourdenx, J. Madrigal-Matute, S. Tiano, A. Diaz, B. A. Bartholdy, B. Will, A. M. Cuervo, Proteome-wide analysis of chaperone-mediated autophagy targeting motifs. *PLOS Biol.* **17**, e3000301 (2019).

90. S. Truckenbrodt, A. Viplav, S. Jähne, A. Vogts, A. Denker, H. Wildhagen, E. F. Fornasiero, S. O. Rizzoli, Newly produced synaptic vesicle proteins are preferentially used in synaptic transmission. *EMBO J.* **37**, 1–24 (2018).

91. A. Andersson, J. Remnestål, B. Nellgård, H. Vunk, D. Kotol, F. Edfors, M. Uhlén, J. M. Schwenk, L. L. Ilag, H. Zetterberg, K. Blennow, A. Månberg, P. Nilsson, C. Fredolini, Development of parallel reaction monitoring assays for cerebrospinal fluid proteins associated with Alzheimer's disease. *Clin. Chim. Acta* **494**, 79–93 (2019).

92. A. Mendsaikhan, I. Tooyama, J. P. Bellier, G. E. Serrano, L. I. Sue, L. F. Lue, T. G. Beach, D. G. Walker, Characterization of lysosomal proteins progranulin and prosaposin and their interactions in Alzheimer's disease and aged brains: Increased levels correlate with neuropathology. *Acta Neuropathol. Commun.* **7**, 215 (2019).

93. J. Vesa, E. Hellsten, L. A. Verkruyse, L. A. Camp, J. Rapola, P. Santavuori, S. L. Hofmann, L. Peltonen, Mutations in the palmitoyl protein thioesterase gene causing infantile neuronal ceroid lipofuscinosis. *Nature* **376**, 584–587 (1995).

94. T. Kon, K. Tanji, F. Mori, A. Kimura, A. Kakita, K. Wakabayashi, Immunoreactivity of myelin-associated oligodendrocytic basic protein in Lewy bodies. *Neuropathology* **39**, 279–285 (2019).

95. G. U. Höglinger, N. M. Melhem, D. W. Dickson, P. M. A. Sleiman, L. S. Wang, L. Klei, R. Rademakers, R. De Silva, I. Litvan, D. E. Riley, J. C. Van Swieten, P. Heutink, Z. K. Wszolek, R. J. Uitti, J. Vandrovcova, H. I. Hurtig, R. G. Gross, W. Maetzler, S. Goldwurm, E. Tolosa, B. Borroni, P. Pastor, PSP Genetics Study Group, L. B. Cantwell, M. R. Han, A. Dillman, M. P. Van Der Brug, J. R. Gibbs, M. R. Cookson, D. G. Hernandez, A. B. Singleton, M. J. Farrer, C. E. Yu, L. I. Golbe, T. Revesz, J. Hardy, A. J. Lees, B. Devlin, H. Hakonarson, U. Müller, G. D. Schellenberg, Identification of common variants influencing risk of the tauopathy progressive supranuclear palsy. *Nat. Genet.* **43**, 699–705 (2011).

96. R. J. O'Brien, P. C. Wong, Amyloid precursor protein processing and Alzheimer's disease. *Annu. Rev. Neurosci.* **34**, 185–204 (2011).

7. Y. Cheng, N. X. Cawley, T. Yanik, S. R. K. Murthy, C. Liu, F. Kasikci, D. Abebe, Y. P. Loh, A human carboxypeptidase E/NF-α1 gene mutation in an Alzheimer's disease patient leads to dementia and depression in mice. *Transl. Psychiatry* **6**, e973 (2016).

98. V. Plá, S. Paco, G. Ghezali, V. Ciria, E. Pozas, I. Ferrer, F. Aguado, Secretory sorting receptors carboxypeptidase E and secretogranin III in amyloid β-associated neural degeneration in Alzheimer's disease. *Brain Pathol.* **23**, 274–284 (2013).

99. I. Begcevic, M. Tsolaki, D. Brinc, M. Brown, E. Martinez-Morillo, I. Lazarou, M. Kozori, F. Tagaraki, S. Nenopoulou, M. Gkioka, E. Lazarou, B. Lim, I. Batruch, E. P. Diamandis, Neuronal pentraxin receptor-1 is a new cerebrospinal fluid biomarker of Alzheimer's disease progression. *F1000Res.* **7**, 1012 (2018).

100. M. F. Xiao, D. Xu, M. T. Craig, K. A. Pelkey, C. C. Chien, Y. Shi, J. Zhang, S. Resnick, O. Pletnikova, D. Salmon, J. Brewer, S. Edland, J. Wegiel, B. Tycko, A. Savonenko, R. H. Reeves, J. C. Troncoso, C. J. McBain, D. Galasko, P. F. Wegiel, NPTX2 and cognitive dysfunction in Alzheimer's disease. *eLife* **6**, 1–27 (2017).

101. T. Nakaya, M. Maragkakis, Amyotrophic lateral sclerosis associated FUS mutation shortens mitochondria and induces neurotoxicity. *Sci. Rep.* **8**, 1–15 (2018).

102. S. Ravanidis, E. Doxakis, RNA-binding proteins implicated in mitochondrial damage and mitophagy. *Front. Cell Dev. Biol.* **8**, 372 (2020).

103. A. Vagnoni, M. S. Perkinton, E. H. Gray, P. T. Francis, W. Noble, C. C. J. Miller, Calsyntenin-1 mediates axonal transport of the amyloid precursor protein and regulates aβ production. *Hum. Mol. Genet.* **21**, 2845–2854 (2012).

04. P. Hallock, M. A. Thomas, Integrating the Alzheimer's disease proteome and transcriptome: A comprehensive network model of a complex disease. *OMICS* **16**, 37–49 (2012).

105. D. R. Gabrych, V. Z. Lau, S. Niwa, M. A. Silverman, Going too far is the same as falling short†: Kinesin-3 family members in hereditary spastic paraplegia. *Front. Cell. Neurosci.* **13**, 419 (2019).

106. C. Vidoni, C. Follo, M. Savino, M. A. B. Melone, C. Isidoro, The role of cathepsin D in the pathogenesis of human neurodegenerative disorders. *Med. Res. Rev.* **36**, 845–870 (2016).

107. J. Li, F. Cao, H.-l. Yin, Z.-j. Huang, Z.-t. Lin, N. Mao, B. Sun, G. Wang, Ferroptosis: Past, present and future. *Cell Death Dis.* **11**, 88 (2020).

108. M. Vacher, T. Porter, V. L. Villemagne, L. Milicic, M. Peretti, C. Fowler, R. Martins, S. Rainey-Smith, D. Ames, C. L. Masters, C. C. Rowe, J. D. Doecke, S. M. Laws, Validation of a priori candidate Alzheimer's disease SNPs with brain amyloid-beta deposition. *Sci. Rep.* **9**, 1–8 (2019).

109. D. Peterson, C. Munger, J. Crowley, C. Corcoran, C. Cruchaga, A. M. Goate, M. C. Norton, R. C. Green, R. G. Munger, J. C. S. Breitner, K. A. Welsh-Bohmer, C. Lyketsos, J. Tschanz, J. S. K. Kauwe, Variants in PPP3R1 and MAPT are associated with more rapid functional decline in Alzheimer's disease: The Cache County Dementia Progression Study. *Alzheimers Dement.* **10**, 366–371 (2014).

110. M. N. Perkovic, D. S. Strac, L. Tudor, M. Konjevod, G. N. Erjavec, N. Pivac, Catechol-O-methyltransferase, cognition and Alzheimer's disease. *Curr. Alzheimer Res.* **15**, 408–419 (2017).

111. J. P. M. Finberg, Inhibitors of MAO-B and COMT: Their effects on brain dopamine levels and uses in Parkinson's disease. *J. Neural Transm.* **126**, 433–448 (2019).

112. H. X. Deng, W. Chen, S. T. Hong, K. M. Boycott, G. H. Gorrie, N. Siddique, Y. Yang, F. Fecto, Y. Shi, H. Zhai, H. Jiang, M. Hirano, E. Rampersaud, G. H. Jansen, S. Donkervoort, E. H. Bigio, B. R. Brooks, K. Ajroud, R. L. Sufit, J. L. Haines, E. Mugnaini, M. A. Pericak-Vance, T. Siddique, Mutations in UBQLN2 cause dominant X-linked juvenile and adult-onset ALS and ALS/dementia. *Nature* **477**, 211–215 (2011).

113. J. E. Gerson, H. Linton, J. Xing, A. B. Sutter, F. S. Kakos, J. Ryou, N. Liggans, L. M. Sharkey, N. Safren, H. L. Paulson, M. I. Ivanova, Shared and divergent phase separation and aggregation properties of brain-expressed ubiquilins. *Sci. Rep.* **11**, 287 (2021).

114. H.-J. Chen, J. C. Mitchell, S. Novoselov, J. Miller, A. L. Nishimura, E. L. Scotter, C. A. Vance, M. E. Cheetham, C. E. Shaw, The heat shock response plays an important role in TDP-43 clearance: Evidence for dysfunction in amyotrophic lateral sclerosis. *Brain* **139**, 1417–1432 (2016).

115. A. K. Whitbread, A. Masoumi, N. Tetlow, E. Schmuck, M. Coggan, P. G. Board, Characterization of the omega class of glutathione transferases. *Methods Enzymol.* **401**, 78–99 (2005).

116. Y. J. Li, S. A. Oliveira, P. Xu, E. R. Martin, J. E. Stenger, C. R. Scherzer, M. A. Hauser, W. K. Scott, G. W. Small, M. A. Nance, R. L. Watts, J. P. Hubble, W. C. Koller, R. Pahwa, M. B. Stern, B. C. Hiner, J. Jankovic, C. G. Goetz, F. Mastaglia, L. T. Middleton, A. D. Roses, A. M. Saunders, D. E. Schmechel, S. R. Gullans, J. L. Haines, J. R. Gilbert, J. M. Vance, M. A. Pericak-Vance, Glutathione S-transferase omega-1 modifies age-at-onset of Alzheimer disease and Parkinson disease. *Hum. Mol. Genet.* **12**, 3259–3267 (2003).

117. L. Kay, I. S. Pienaar, R. Cooray, G. Black, M. Soundararajan, Understanding miro GTPases: Implications in the treatment of neurodegenerative disorders. *Mol. Neurobiol.* **55**, 7352–7365 (2018).

118. F. Zhang, W. Wang, S. L. Siedlak, Y. Liu, J. Liu, K. Jiang, G. Perry, X. Zhu, X. Wang, Miro1 deficiency in amyotrophic lateral sclerosis. *Front. Aging Neurosci.* **7**, 100 (2015).

119. D. Grossmann, C. Berenguer-Escuder, A. Chemla, G. Arena, R. Krüger, The emerging role of RHOT1/Miro1 in the pathogenesis of Parkinson's disease. *Front. Neurol.* **11**, 587 (2020).

120. S. Quintremil, F. Medina Ferrer, J. Puente, M. Elsa Pando, M. Antonieta Valenzuela, in *Neurons—Dendrites and Axons* (IntechOpen, 2019).

121. E. Cantó, M. Tintoré, L. M. Villar, E. Borrás, J. C. Álvarez-Cermeño, C. Chiva, E. Sabidó, A. Rovira, X. Montalban, M. Comabella, Validation of semaphorin 7A and ala-β-hisdipeptidase

as biomarkers associated with the conversion from clinically isolated syndrome to multiple sclerosis. *J. Neuroinflammation* **11**, 181 (2014).

122. S. Mueller-Steiner, Y. Zhou, H. Arai, E. D. Roberson, B. Sun, J. Chen, X. Wang, G. Yu, L. Esposito, L. Mucke, L. Gan, Antiamyloidogenic and neuroprotective functions of cathepsin B: Implications for Alzheimer's disease. *Neuron* **51**, 703–714 (2006).
