# Supplementary material for: Protein lifetimes in aged brains reveal a proteostatic adaptation linking physiological aging to neurodegeneration
Source: Sci Adv. 2022 May 20;8(20):eabn4437. doi: 10.1126/sciadv.abn4437 (PMC9122331; doi:10.1126/sciadv.abn4437)
Supplement: Supplementary file 2 — Tables S1 to S7 [file sciadv.abn4437_tables_s1_to_s7.zip › sciadv.abn4437_table_s4.docx]

**Table S4:**

**Relatively longer-lived proteins in the aged mouse brain are connected to neurodegenerative disease (NDD)**

| **Name** | **Avg log_2_FC 21m/5m** | **NDD** | **Protein function** | **Connections to NDD** | **Ref.** |
| --- | --- | --- | --- | --- | --- |
| Psap | 1.53 | AD | lysosomal protein | CSF pre-clinical biomarker of AD; increased levels correlate with neuropathology and A-β | (*91*, *92*) |
| Ppt1 | 1.51 | NCL (CLN1) | lysosomal enzyme | Mutations in the CLN1 gene cause infantile neuronal ceroid lipofuscinosis | (*64*, *93*) |
| Sorl1 | 1.20 | AD | sorting receptor | Regulating A-β production and trafficking | (*57*, *58*) |
| Mobp | 0.87 | supranuclear palsy | myelin sheath | Mutations are risk factor for tauopathies and AD; MOBP accumulates in Lewy Bodies | (*94*, *95*) |
| App | 0.68 | AD | surface receptor | A-β precursor | (*96*) |
| Cpe | 0.66 | AD | exopeptidase, sorting receptor | Failure of regulated secretion is linked to A-β-associated neurodegeneration in AD patients and mouse models | (*97*, *98*) |
| Nptx1 | 0.64 | AD | neurodevelopment, synapse formation | Excitatory synapse dysfunction connected to cognitive decline; CSF biomarker of AD disease progression | (*99*, *100*) |
| Csde1 | 0.60 | ALS | RNA-binding protein | FUS mutants alter the RNA landscape, affecting Csde1 levels and thus mitochondrial size and function; Csde1 also regulates stress-granule formation | (*93*, *101*, *102*) |
| Clstn1 | 0.58 | AD | axonal transport | Influences APP transport, processing and A-β production | (*59*, *103*) |
| Mapk8ip3 | 0.57 | AD | vesicle transport | Involved in the clathrin-mediated endocytosis and trafficking of APP | (*104*) |
| Kif1a | 0.57 | AD | microtubule motor protein | BACE1 is a cargo of Kif1a and its improper localization influences APP processing and A-β load | (*105*) |
| Ide | 0.56 | AD | protease | IDE1 is involved in amyloidogenic-protein elimination | (*60*) |
| Ctsd | 0.49 | AD | lysosomal enzyme | Ctsd is essential for degradation of unfolded or oxidized proteins | (*61*, *106*) |
| Fth1 | 0.48 | AD, PD | iron homeostasis | Iron homeostasis and ferroptosis are heavily implicated in several NDD | (*33*, *107*) |
| Ppp3r1 | 0.44 | AD | synaptic activity | Mutations is Ppp3r1 have been linked to tau accumulation and AD disease progression | (*108*, *109*) |
| Comt | 0.44 | PD, AD | dopamine metabolism | COMT inhibitors are used as PD medication and might slow cognitive decline also in AD | (*110*, *111*) |
| Ubqln2 | 0.43 | ALS | protein degradation | Ubqln2 mutations cause impaired protein degradation and neurodegeneration in ALS | (*112*, *113*) |
| Dnajb2 | 0.41 | ALS | heat shock protein | DNAJB2 is a potent anti-aggregator of TDP-43 and its function is compromised in ALS | (*114*) |
| Gsto1 | 0.40 | AD, PD | glutathione transferase | Polymorphisms in Gsto1 are linked to age at onset in AD and PD | (*115*, *116*) |
| Rhot2 | 0.39 | PD, AD, ALS | mitochondrial rho GTPase | Rhot2 aka Miro1 is linked to compromised mitochondrial transport, quality control and mitophagy in PD, AD and ALS | (*117*–*119*) |
| Sema7a | 0.37 | MS, PD | neuron plasticity | Sema7a is involved in several neuron- and neuroimmuno processes and is implicated in MS | (*120*, *121*) |
| Ctsb | 0.34 | AD | lysosomal enzyme | Ctsb is essential for degradation of unfolded or oxidized proteins, among them A-β | (*61*, *122*) |
